# tACS-combined motor training for the rehabilitation of the upper limb in children and adolescents with cerebral palsy: A randomized, sham-controlled trial protocol

**DOI:** 10.1371/journal.pone.0331360

**Published:** 2025-09-03

**Authors:** Viola Oldrati, Andrea Ciricugno, Renato Borgatti, Simona Orcesi, Elisa Fazzi, Jessica Galli, Verusca Gasparroni, Luigi Piccinini, Cristina Maghini, Zaira Cattaneo, Maria Arioli, Cosimo Urgesi, Alessandra Finisguerra

**Affiliations:** 1 Scientific Institute, IRCCS E. Medea, Bosisio Parini (LC), Italy; 2 Department of Brain and Behavioral Sciences, University of Pavia, Pavia, Italy; 3 IRCCS C. Mondino Foundation, Pavia, Italy; 4 Department of Clinical and Experimental Sciences, University of Brescia, Brescia, Italy; 5 Unit of Child Neurology and Psychiatry, ASST Spedali Civili of Brescia, Brescia, Italy; 6 Department of Human and Social Sciences, University of Bergamo, Bergamo, Italy; 7 Department of Human and Social Sciences, Universitas Mercatorum, Rome, Italy; Beijing Sport University, CHINA

## Abstract

**Background:**

Children with cerebral palsy (CP) commonly face gross motor function impairments and manual dexterity deficits, significantly affecting their activity level and independence and, ultimately, quality of life. Rehabilitation often targets improving manual dexterity and activity levels, but standard therapies have limited efficacy. Hence, exploring novel methods to enhance upper limb functionality is crucial. Transcranial alternating current stimulation (tACS), by delivering currents oscillating at specific frequencies syncing with the brain’s electrical rhythms, has been demonstrated to modulate neural oscillations and motor behavior.

**Method:**

This randomized, double-blind, sham-controlled, pre-post test study involves 44 children and adolescents (6–17 yo) with CP treated in pairs, which will be randomly allocated to the experimental or control group receiving, respectively, active or sham fronto-cerebellar tACS delivered at the individual gamma frequency. After tACS, both groups will undergo bimanual training, including lower extremities (HABIT-ILE). Primary outcome measures will include the Assisting Hand Assessment, Box and Block Test, and a Visuomotor Task administered via computer for manual visuomotor control evaluation. Secondary outcomes will encompass the Children’s Hand Experience Questionnaire, Canadian Occupational Performance Measure, Melbourne Assessment of Unilateral Upper Limb Function, Gross Motor Function Measure, Vineland version 2, Pediatric Quality of Life Inventory, and EEG power recorded in fronto-central regions at rest before (at T0), soon after (at T1), and 3 months after the end of (T2) the training. Safety and tolerability will be assessed by pre- and post-tACS recordings of oxygen saturation and heart rate, along with self-report questionnaires on sensations and side-effects.

**Discussion:**

This study investigates whether an intensive HABIT-ILE program combined with fronto-cerebellar gamma tACS can boost training effects on manual dexterity in children and adolescents with CP, while ensuring safety and tolerability throughout the intervention period.

**Trial registration:**

ClinicalTrials.gov NCT06372041

## Introduction

Cerebral Palsy (CP) encompasses a range of permanent movement and posture disorders, leading to activity limitations and often accompanied by disturbances in sensation, perception, cognition, communication, and behavior. These challenges profoundly affect the social participation and quality of life of both patients affected with CP and their families, persisting into adulthood [1,2], with an overall prevalence of 2.11 per 1000 live births across the globe [3,4]. Early encephalopathy in the pediatric population, whether of vascular, infectious, or neuro-inflammatory origin, can be a causative factor [5]. Depending on the location of brain lesions, CP may manifest as either bilateral or unilateral deficits. The functionality of the upper limbs significantly influences a child’s ability to perform self-care tasks and engage in leisure and educational activities. Children with CP often encounter frustration when attempting everyday two-handed tasks and exhibit greater negative reactions to failure compared to their typically developing peers [6].

Evidence-based recommendations advocate for an early, intensive and personalized management program developed collaboratively with the child or young person and their parents or caregivers, focusing on individualized goals (see [7] for a review). Such programs, typically involving physical therapy (comprising physiotherapy and/or occupational therapy), should be tailored to meet the unique needs of the patient [8–11]. Its objectives include enhancing skill development, improving functions, and facilitating participation in daily activities. Consistent with these recommendations, a systematic review conducted by Jackman et al. in 2020 [2] highlighted that interventions emphasizing the repetition of isolated movements are less effective compared to those centered around goal achievement and skill acquisition, which can be integrated into a child’s daily routine. Accordingly, in contrast to passive, bottom-up therapeutic approaches, task-specific interventions have emerged as effective treatments for children with unilateral CP [12,13].

The hand-arm bimanual intensive therapy including lower extremities (HABIT-ILE), which integrates upper and lower extremity engagement, has demonstrated positive results in randomized controlled trials involving children with unilateral CP [14–18]. HABIT-ILE is characterized by its intensive nature, often involving several hours of therapy per day over a concentrated period, which is believed to be essential for driving neuroplasticity changes and maximizing functional gains. Therapy sessions typically involve engaging the child in tasks that are relevant to their daily life and functional goals, including activities tailored to the child’s interests and needs. Despite the recognized importance of early and intensive interventions for treating CP, there remains a pressing need to identify novel interventions that target brain plasticity to optimize treatment outcomes.

Although there is currently no cure for CP, emerging research suggests that Non-invasive Brain Stimulation (NIBS), alone or in combination with rehabilitation regimens, holds promise for mitigating motor impairments in affected patients. NIBS techniques, such as Transcranial Magnetic Stimulation (TMS) and Transcranial Electrical Current Stimulation (tES), have shown potential in modulating the primary motor cortex and yielding positive effects on motor functions. While most studies utilizing NIBS have focused on adults with stroke [19,20], there is evidence suggesting that their clinical utility can extend to children with brain injuries [21–24].

Among tES techniques, transcranial Alternating Current Stimulation (tACS) delivers weak alternating electrical currents at specific frequencies, which penetrate through the scalp and modulate neuronal activity and behavior [25]. Initially, it was hypothesized that tACS effects depended on entraining neural populations by aligning ongoing brain rhythms with an external alternating current [26]; though, more recent research emphasizes the complex interactions between the applied sinusoidal current and ongoing neural activity [27]. Despite ongoing efforts to clarify its physiological mechanisms of action, there has been a surge in clinical trials investigating the potential benefits of tACS on neurological symptoms [28]. Indeed, rhythmic brain oscillations play a crucial role in various physiological and behavioral processes, and abnormalities in these oscillatory patterns are observed in neurological disorders.

Notably, gamma oscillatory activity (from 40 to 80 Hz) has attracted attention by its putative role in motor control and movement execution [29,30]. Motor gamma oscillations have been recorded during both basic movements, like finger movements [31], and more complex actions, like motor imagery [32] and interpersonal interactions [33]. Accordingly, local motor gamma activity is typically detected in the primary motor cortex (M1) [31,34] and accessory motor regions, such as the supplementary motor area (SMA) [35,36], the premotor cortex (PMC) [36,37], as well as areas proximal to the frontal cortex [38]. Interestingly, research indicates a deviant gamma response in children with CP in comparison to healthy peers [29,39,40].

Ongoing research is delving into the impact of gamma tACS applied over the cerebellum on motor functions [41,42]. Indeed, the cerebellum is a central hub within the motor learning network [43,44], and its firing patterns at 50 Hz coincide with the basal activity of Purkinje cells [45], a unique type of neuron specific to the cerebellar cortex. In their study, Naro et al. [46] employed a paired tACS-TMS approach to investigate the impact of cerebellar tACS on cortical excitability, assessed through M1 stimulation via TMS. The researchers discovered that applying 50-Hz tACS over the right cerebellum increased the amplitude of TMS-induced motor evoked potentials (MEPs) and hastened the execution of simple movements. Similarly, a recent study [47] reported that 50-Hz tACS over the cerebellum heightened motor cortex excitability when applied at rest, though not during movement execution. Moreover, the stimulation improved reaction times for manual motor sequences when administered during task execution. In keeping with this finding, Giustiniani et al. [48] reported significant behavioral enhancements in the acquisition of implicit manual motor sequences following 50-Hz tACS over the right cerebellum. Conversely, Wessel et al. [49] found no discernible effects of 50-Hz cerebellar tACS on motor performance or measures of intracortical inhibitory and facilitatory neurotransmission. Likewise, Spampinato et al. [50] failed to observe significant effects of 50-Hz cerebellar tACS on both motor performance and measures of corticospinal excitability.

Expanding beyond targeting the cerebellum alone, Miyaguchi and colleagues [51] explored the effects of 70-Hz tACS over both M1 and the right cerebellar hemisphere. They observed improved motor performance in a force task among poor performers compared to sham stimulation. This effect, later replicated and consistent regardless of stimulation intensity [52], possibly indicated tACS-induced enhancement of functional synchronization between the two regions [53]. Despite discrepancies in study findings, likely stemming from methodological variations, this evidence encourages the exploration of the potential of tACS as a low-cost clinical tool to treat motor impairments.

Leveraging these findings alongside the promising effects of gamma tACS on motor performance and neuroplasticity, this study primarily aims to assess the effectiveness of a fronto-cerebellar tACS protocol integrated with an intensive training (HABIT-ILE) in improving manual visuomotor control in a group of pediatric CP patients. Once recruited, patients will be divided into two groups: one group will undergo the rehabilitation regimen alongside active tACS application, while the other will receive a sham stimulation protocol in combination with the standard rehabilitation regimen. Specifically, we hypothesize that: I) patients will show an improvement both in primary and secondary outcomes, including functional motor abilities, goal-oriented skills and psychological adjustment, following the HABIT-ILE training; II) patients treated with active tACS + HABIT-ILE will show a greater and more lasting improvement compared to patients in the sham tACS + HABIT-ILE group, suggesting that tACS may enhance training outcomes by optimizing brain receptiveness to motor learning, thereby potentially boosting its effects.

In this study, we will investigate a novel fronto-cerebellar tACS montage. The cerebellum has emerged as a key target for NIBS due to its central role in both motor and non-motor functions. Beyond its functional importance, the cerebellum’s extensive connectivity with both cortical and subcortical regions – particularly via the dentate-thalamus-cortical pathway – enables it to modulate distant brain areas, even those affected by injury or disease [54]. These features, combined with its function as an adaptive controller, make the cerebellum an ideal candidate for neuromodulation in a wide range of neurological conditions [55]. Building upon the ‘binding theory’, which posits that neural populations stimulated in distinct cortical regions synchronize with gamma band oscillations, targeting specific areas at this frequency may enhance inter-cortical neural networks [56]. Given the cerebellum’s ‘closed-loop’ connections with the prefrontal cortex thought to underlie the integration of sensorimotor information [57], it is hypothesized that a fronto-cerebellar tACS montage could influence long-range connections between these regions. Indeed, a recent study [58] in healthy adults has shown that stimulating the fronto-cerebellar circuits with the same tACS montage may enhance motor performance and modulate inhibitory cortical dynamics, supporting its use as a precision neuromodulation tool in clinical settings. Recent research has also revealed changes in activation patterns following HABIT-ILE intervention in children with CP, which encompassed a comprehensive network spanning the fronto-parietal cortex and cerebellum [59], all together these findings support the exploration of the effectiveness of such montage.

Moreover, previous findings suggested that weak exogenous electric stimulation fails to induce a frequency shift in ongoing oscillations unless matched to the endogenous oscillation frequency, implying that tACS may primarily enhance, rather than override, intrinsic network dynamics [60]. Therefore, we will tailor the stimulation protocol to each patient by extracting their individual gamma frequency (IGF) and applying it during the intervention.

## Methods

### Study design

This represents one of the two randomized clinical trials (RCTs) of the project “Bottom-up and tOp-down neuromOdulation of motor plaSTicity in cerebral palsy” (BOOST; FRRB 3438840). The study employs a randomized, double-blind, sham-controlled, pre-post-test design with 44 children diagnosed with CP. Participants will undergo an intensive HABIT-ILE training in a motivating environment, receiving either active or sham IGF tACS stimulation. Medical doctors will contact and enroll eligible patients willing to participate. Of note, patients will be treated in pairs, with a matching of motor deficit severity, IQ, or age, by meeting at least two out of these three criteria. Each pair will be assigned randomly to either the active or sham group in a 1:1 allocation ratio, using a computer-generated blocked randomization process carried out by independent personnel of the sponsor institute not directly engaged in the study.

Randomization will be stratified by IQ (two levels: 50–70 and >70), age (two levels: 6–11 years; 12–17 years) or MACS level (two levels: low to middle including I and II levels and middle to high including II and III levels). Priority will be given to stratification based on MACS level, as this variable is most directly related to motor function, which represents the primary focus of the intervention. The following hierarchical strategy will be implemented: participants will be first grouped based on MACS level, distinguishing between “low to middle” (levels I and II) and “middle to high” (levels II and III). Individuals with MACS level II will be allocated to the low-to-middle or to the middle-to-high according to their IQ level. Within each MACS stratum, we will attempt secondary stratification by age and IQ when feasible, given the distribution of participants. In cases where mixed pairs occur within a MACS stratum (e.g., a participant classified as MACS I is paired with a participant classified as MACS II), these pairs will be counterbalanced across the real and sham stimulation groups to ensure balanced allocation.

**Fig 1:**
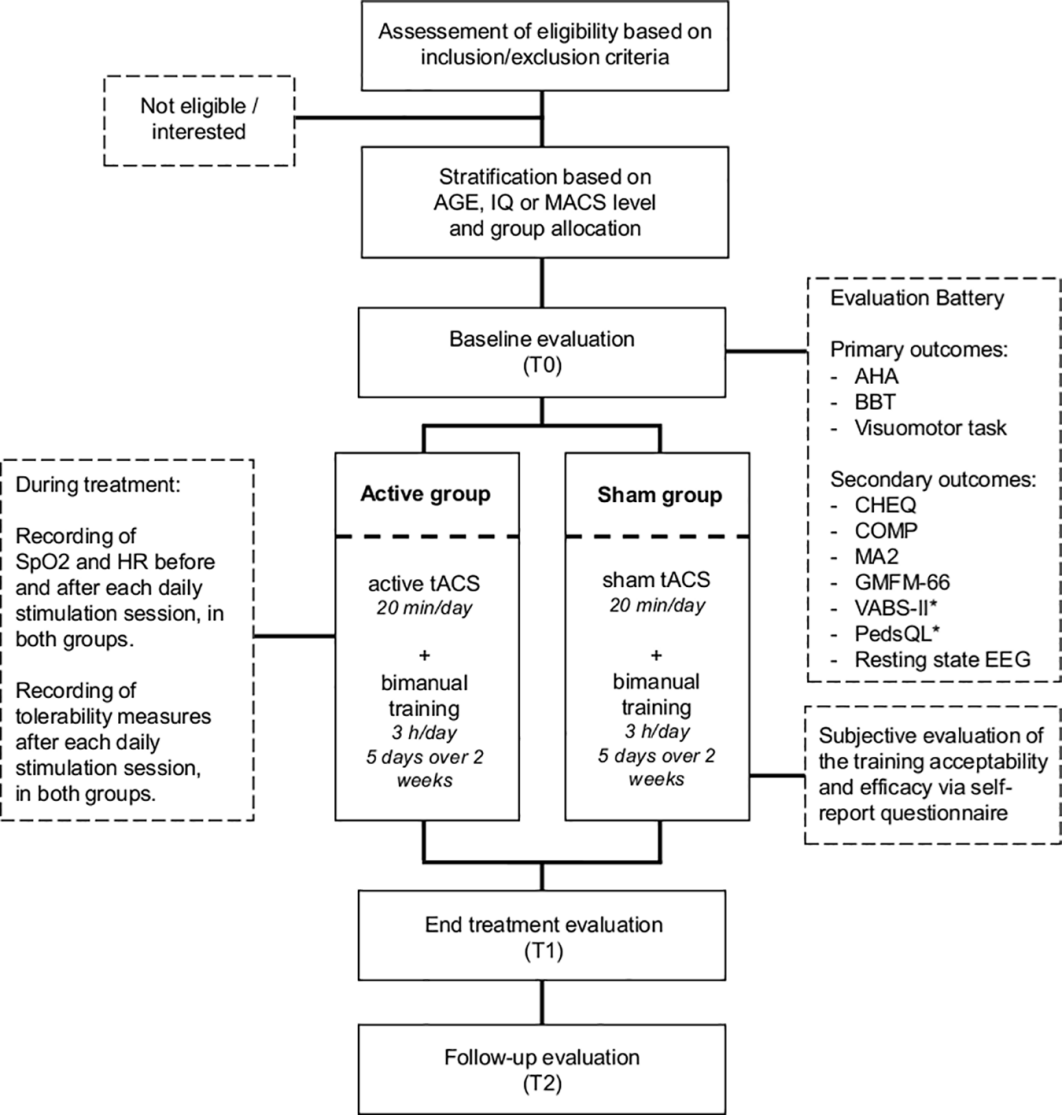
Flow chart of patient inclusion.

Staff conducting pre/post assessments, therapists leading the training and researchers analyzing data will be blinded, whereas the personnel administering the stimulation and the project administrators will not be blinded. The personnel administering the stimulation will be the only person in the room aware of the correspondence between a specific code and stimulation protocol and will leave when the stimulation (either active or sham) is over, thus preventing any influence on training administration. Of note, the sham stimulation will be undistinguishable from active stimulation for both participants and therapists (see below), ensuring that the therapist conducting the training remains unaware of the treatment allocation. Unblinding should only occur in rare cases where knowledge of the treatment is crucial for managing the patient, such as in case of adverse events. Assessments will occur at baseline (T0; from one to two days before the beginning of the training), post-treatment (T1; from one to two days after the end of the training), and three months’ follow-up (T2). All primary and secondary outcomes will be assessed at each time point, except for vital parameters and questionnaires on stimulation-induced sensations, which will be evaluated before and after each daily stimulation session, and questionnaires gauging psychological and functional adaptation, which will be administered solely at T0 and T2 (see Fig 1).

The overall RCT will be structured as follows. Prior to the beginning of the training (T0), the procedure requires the administration of all clinical assessment measures. Additionally, patients will be asked to perform for 10 minutes an ad-hoc computer based Visuomotor task (please refer to the primary outcome paragraph for more detailed description). Cortical rhythms will be recorded by electroencephalogram (EEG) both before (during rest) and during the execution of the Visuomotor task. Analysis of the resting-state EEG will be conducted to derive the IGF, which will be matched to each patient’s specific stimulation frequency delivered through tACS during the HABIT-ILE training.

The intensive treatment will be conducted at Scientific Institute IRCCS E. Medea (study sponsor), Fondazione Mondino IRCCS, and ASST Spedali Civili di Brescia. All institutes are recruitment centers. Recruitment began on April 15, 2024, and is expected to conclude on January 14, 2026. Data collection will continue through the follow-up period, ending three months after the last participants are enrolled, in March 2026. Results are expected to be available by May 2026, a few months after data collection is completed.

### Patient selection

Children and adolescents with a diagnosis of CP meeting the following criteria will be eligible: aged 6–17 years, demonstrating clinical signs of unilateral or bilateral upper limb deficits with a minimum 25% difference in performance between the more affected and less affected hand on the Box and Block test; confirmed diagnosis via Magnetic Resonance Imaging according to Surveillance of Cerebral Palsy (SCPE) criteria; Manual Ability Classification System (MACS) level between I-III; Gross Motor Function Classification System (GMFCS) level between I-III; Visual Function Classification System (VFCS) level between I-III; capable of following instructions and completing age-appropriate assessments (IQ > 50). Exclusion criteria include cochlear implant, cardiac pacemaker, neurostimulators, ventriculoperitoneal shunt, clips, fragments, or metal splinters in the brain or skull; motor deficits not of perinatal origin (e.g., head trauma); severe visual impairment hindering treatment or testing; spasticity treatments or upper limb functional surgery within the past 6 months or planned during the duration of the study; uncontrolled epileptic seizures in the last 2 years.

### Outcomes

Primary outcomes comprise two validated measures of spontaneous use and manual dexterity of the more affected hand – Assisting Hand Assessment (AHA) and Box and Block Test (BBT) – and a computer-administered, customized visuomotor task for assessing manual visuomotor control. Secondary outcomes include a thorough evaluation of motor skills, functional adaptation, and quality of life. Further details regarding both primary and secondary outcomes are provided in the following paragraphs. A schematic depiction of the schedule of enrolment, intervention, and assessment can be found in the supplementary material (S1 Fig.).

#### Primary outcomes.

- The Assisting Hand Assessment (AHA) quantifies the assistance provided by the more affected hand to the less affected hand during bimanual activities. This observation-based, criterion-referenced assessment highlights a person’s typical performance, emphasizing practical functionality over maximal capacity, and serves as a reliable measure of change over time. The scale comprises 20 items, scored on a 4-point Likert scale, from 1 to 4, with the total score indicating how well the more affected hand is used as an assisting hand. The results are converted to logits by a Rasch analysis on a 0-to-100 scale, with higher scores suggesting better use. This assessment has been shown to be responsive, reliable and valid for use in children with CP [61].
- The Box and Block test, designed to measure manual dexterity in CP [62,63], is quick, simple, and cost-effective. It involves a box with a partition in the middle placed on a table, with blocks on one side of the partition. The score is the number of blocks transported within a minute with the less and the more affected hand. The BBT provides a reliable and objective measurement of manual dexterity, making it valuable for evaluating functional outcomes and monitoring progress in rehabilitation programs over a short period of time [64].
- The Visuomotor, pc-administered ad-hoc task was created for the evaluation of manual visuomotor control. The task involves a mouse click-and-drag operation where an object appears at the center of the screen. The objective is to drag and drop the object to the location indicated by a previously presented arrow, pointing towards a target object within a configuration of objects. Participants are required to focus on the arrow’s direction, swiftly and accurately moving the central object to its designated target location. This task enables the measurement of movement time (in milliseconds; consisting in the time necessary to move the object in the target position); precision error (calculated as the distance, in pixels between the drop position of the object and the actual target position); the proportion of overtime errors (the percentage of trials in which responses are too slow). Smaller values of all the three variables point to better performance.

#### Secondary outcomes.

- The Children’s Hand-use Experience Questionnaire (CHEQ) comprises bimanual activities that are rated on scales measuring perceived efficacy of the activity, amount of assistance needed, and the child’s satisfaction with their performance. The scoring process involves assessing the responses provided by the child or adolescent to these items, which are then used to gauge their hand-use experience in daily tasks. The result in the report consists in a summary of the ratings on a 0-to-100 scale, with higher scores suggesting better performance/satisfaction [65].
- The Canadian Occupational Performance Measure (COPM) is a client-centered, semi-structured interview used in occupational therapy to evaluate patients’ outcomes in self-care, productivity, and leisure [66]. It is scored based on client’s self-reported ratings of perceived performance and satisfaction with their performance in identified areas of occupation. Each area of occupation is rated on a scale from 1 to 10, with 1 representing the lowest level of performance or satisfaction and 10 representing the highest.
- The Melbourne Assessment-2 (MA2) will be used to evaluate the unimanual performance of both the more and less affected hand according to movement range, accuracy, dexterity and fluency. It consists of 14 test items that require children to interact with simple objects, with scoring of each item on a Likert scale. A child’s total raw score for each sub-scale is converted to a percentage of the maximum possible score for that sub-scale, with higher scores indicating better performance. The MA2 is a valid and reliable measure for the assessment of the quality of upper limb movements in children with neurological conditions [67].
- The Gross Motor Function Measure (GMFM–66) [68] is a standardized observational tool composed of 66 items that assess gross motor function. Each item is scored on a 4-point scale. The scores for each item are then summed to obtain a total score, which represents the child’s overall gross motor function. The score will be expressed as a percentage of the maximum possible score, providing a standardized measure of gross motor function ability.
- The Vineland Adaptive Behavior Scale Version 2 (VABS-II) is a tool designed to assess adaptive behavior in individuals from birth to age 90 yo, including open-ended questions to gather in-depth information. This scale is used to assess adaptive skills across 4 domains: communication, daily living skills, socialization and motor skills. It provides 5 scores for each domain and a total score on a 100 ± 15 distribution. It has a proven track record of applications in the evaluation of children with CP [69].
- The Pediatric Quality of Life Inventory (PedsQL, cerebral palsy module) is a widely employed, brief, and standardized self-reporting tool for assessing health-related quality of life in pediatric populations, including children with CP [70]. It is scored based on the responses provided by the child (age-appropriate, self-compiled version) or their parent/caregiver (parent-compiled version) on the questionnaire items, rated on a 5-point Likert scale. The scores are then transformed into a scale ranging from 0 to 100, with higher scores indicating better quality of life.
- Cortical rhythms: cortical rhythms will be recorded using 6 fronto-central EEG electrodes (C1, C2, CZ, FC1, FC2, FCz) at rest (5 minutes) and during the completion of the Visuomotor task (15 minutes), to investigate potential changes in EEG power in the delta, alpha, beta and gamma bands across time-points. This analysis will focus on regions associated with cognitive and motor control, aiming to detect any changes indicative of treatment effects.

To mitigate potential fatigue effects on patient performance, pre-treatment (T0) and post-treatment evaluations (T1 and T2) will be administered across two sessions within a 3-day period for each assessment time point. Each evaluation session requires approximately 2–3 hours: (1) performance-based assessments including the AHA, B&B, GMFM-66, MA2, COPM, and computerized visuomotor tasks, with a maximum duration of 2–3 hours; and (2) questionnaire administration comprising parent- or patient-completed measures (VABS-II, CHEQ, and PedsQL).

#### Safety and tolerability measures.

To assess safety and tolerability to the tACS protocol, vital parameters such as capillary saturation of O2 (SpO2) and heart rate (HR) will be measured during a 2-minute rest condition with a wrist pulse-oximeter at the beginning and end of each daily stimulation session. Patients will also be asked to complete, at the end of the stimulation session, a customized self-perception questionnaire to examine the stimulation-induced sensations (including: tingling, burning, pain, mouth taste, visual sensations like flashes, eyelid movements, others) through a series of Child-friendly Likert scales and the level of discomfort through Visual Analogue Scales.

#### Feasibility and acceptability of the training.

Feasibility of the training will be assessed by considering i) the number of patients who accept to complete the 2-week training and ii) the number of sessions completed per patient. These values, expressed as percentage and mean percentage, will be extrapolated at T1. Higher values will indicate higher feasibility.

Lastly, acceptability will be assessed by asking the child/adolescent and his/her parents’ subjective evaluation of training accessibility and efficacy. The questionnaires will be adapted from the study of Butti and colleagues [71]. Questions like “I find it difficult to motivate my child for doing the training”/ “I find it difficult to start the training”; or “I would suggest this training to other people I know”/ “I think other people I know would enjoy doing this training” will be rated from 1 (completely disagree) to 5 (completely agree). Response will be reversed in the negative items, so that higher scores will suggest more positive evaluation/higher acceptability.

### Transcranial direct current stimulation (tACS)

**Fig 2:**
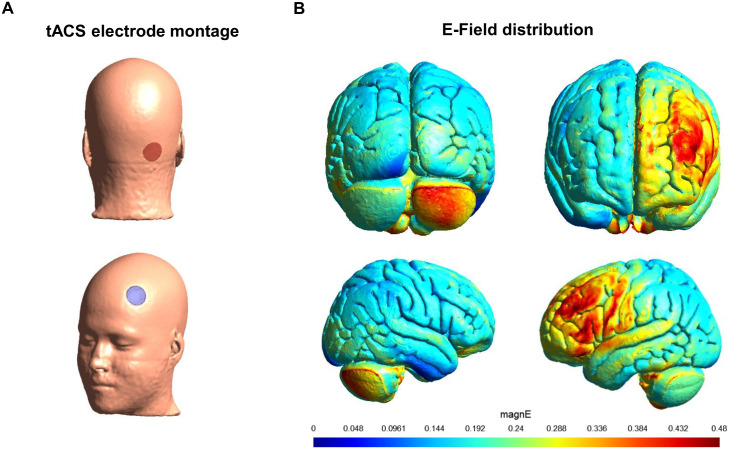
Representation of the estimated electric field induced by fronto-cerebellar IGF tACS using two 8 cm^2^ round-shaped electrodes, the active placed over the right posterior cerebellum (centered 2 cm below and 3 cm to the right of the inion) and the return over F3. The head model was created using fine element modeling on T1- and T2-wighted MRI images of an exemplary subject resulting in a high-resolution tetrahedral head mesh model containing 8 tissue types. Electrodes of 1 mm thickness were simulated to be contained within a 2 mm thick sponge whose size extended the electrode size by 5 mm. All tissues were treated as isotropic. The electrical field E was determined by taking the numerical gradient of the electric potential.

The tACS will be performed by using a CE-marked DC stimulator device (Starstim ®, Neuroelectrics, Barcelona, Spain). It consists in a multi-focal tES-EEG device with 8 channels. The current will be delivered through two round-shaped, saline-soaked surface sponge electrodes (dimension: 8 cm^2^ each), one (return) placed over F3, corresponding to the left prefrontal cortex, and the other (active) over the right cerebellar hemisphere, centered 2 cm below and 3 cm to the right of the inion (Iz) (see Fig 2A), as advised in a computational electric field modeling study [72]. The Starstim neoprene caps include a pre-configured grid of electrode positions based on a subset of the international 10–20 EEG system, allowing for standardized placement of both EEG and neurostimulation electrodes. The F3 location is predefined within the cap’s layout. In contrast, the right cerebellar electrode position will be custom-defined (ad hoc) by manually marking a new site based on the anatomical measures described above to target the cerebellar region, which falls outside the standard 10–20 system coverage. We have opted for a standardized left frontal cortex and right cerebellar montage for all participants, regardless of their lesion’s location or whether it’s unilateral or bilateral. This uniform approach was chosen to prevent the variability that would come from customizing electrode placement for each individual’s lesion. Moreover, recent findings suggest that, even in unilateral CP, motor impairments may be associated with bilateral involvement of motor pathways, including the extrapyramidal system and cerebellum [73].

The stimulation intensity will be set to 1mA, and this value will be gradually reached with a ramping-up phase of 30 secs. At the end of the stimulation time, current will gradually fade out with a similar 30-sec ramp-down phase. The intensity of stimulation has been set in consideration of the thinner skull and lower resistance of younger participants [74]. The frequency of the stimulation will match the IGF identified – within the range 30–80 Hz – during the EEG recording performed at rest. The tACS will be applied for the first 20 minutes of the training, every day of the treatment. In the sham condition, after the initial ramping-up, the stimulation will be switched off. This procedure allows participants to feel the characteristic tingling sensations in the vicinity of the electrodes for a brief period of time, which enhances the plausibility of the sham condition.

A simulation model of the electric field distribution and magnitude generated by the applied montage was performed with SimNIBS 4.1 [75,76], using standard conductivity values (see Fig 2B).

### EEG recording and IGF extraction

EEG recording will be performed using the same tES-EEG device used to deliver tACS (Starstim ®, Neuroelectrics, Barcelona, Spain). EEG data will be recorded at 500 Hz sampling rate, from 6 Ag/AgCl electrodes (C1, C2, Cz, FC1, FC2, FCz) in a 10/10 system, using the right earlobe for both ground and reference. After recording, EEG data will be exported to a format compatible with Matlab® (The Mathworks Inc., 2023) and processed within the EEGLAB signal processing toolbox and custom Matlab® scripts. Continuous EEG data will be filtered using a 0.5-Hz high pass and then a 100-Hz low pass FIR filter.

For each electrode and condition (rest and Visuomotor task execution) the power spectral density (PSD) will be estimated using Welch’s method. The specific frequency within the gamma band (ranging from 30 to 80 Hz), observed during rest and exhibiting the greatest power, will be labeled as the IGF. This frequency will then serve as the stimulation frequency tailored to each individual patient.

### HABIT-ILE program

The HABIT-ILE protocol employs a motor learning-centric approach, integrating problem-solving techniques and meticulously structured practice sessions [14].

The Habit-ILE protocol integrates targeted approaches for enhancing lower extremity function, posture, and interlimb coordination alongside upper extremity exercises. Structured practice sessions involve concurrent upper and lower limb coordination tasks, leveraging neuroplasticity through repeated practice and task escalation within a motivating framework. Tailored interventions are devised based on individual motor abilities, age, interests, and functional goals, with input from parents. Tasks are gradually intensified while ensuring an environment conducive to success yet demanding motor proficiency. Starting at a manageable level, tasks evolve from passive upper extremity involvement to active engagement, encompassing various activities: I) gradually escalating tabletop fine motor activities in a seated position; II) incorporating activities of daily living while seated, standing, or walking and III) introducing gross motor play and physical activities. Progression involves transitioning lower extremity involvement from stable seated activities to dynamic movements like crawling, walking, or running. Advanced upper extremity exercises are initially practiced in stable sitting positions before integrating lower extremity engagement. Throughout sessions, continual feedback enhances performance, with activities structured as games to foster enjoyment and motivation. Therapists prioritize enhancing child-environment interaction and sustaining attention and motivation, ensuring holistic rehabilitation addressing motor, sensory, cognitive, and emotional-relational skills.

While reduced withdrawal and increased motivation may be potential benefits of paired training, this format also appears to support motor learning. Previous research has demonstrated that dyadic practice can be an effective and efficient approach to acquire motor skills in healthy individuals, likely through mechanisms such as observational learning [77]. When applied to clinical contexts, the presence of a peer has been suggested to enhance engagement, normalize perceived effort and encourage persistence. Observing a partner who attempts a task may allow participants to discover alternative strategies and reinforces successful actions, thereby facilitating learning [78]. Training remains individualized according to HABIT-ILE principles, with activities tailored to each participant’s specific goals. When pairs share similar objectives, they engage in common or interactive tasks; when goals differ, training alternates between individual activities while maintaining the shared environment’s benefits.

The total dose of treatment will be 30 hours of HABIT-ILE. The 30 hours will be achieved through a 2-week intensive pair-delivered regimen for 3 hours/day over 10 days. In the first 20 minutes of each session, patients will receive the tACS while performing HABIT-ILE activities at a table position, reducing the impact of possible side effects of tACS (e.g., visual phosphenes) on motor performance.

To ensure protocol adherence, each therapist maintaining the 1:2 ratio will be assisted by a second therapist to monitor training compliance and facilitate the coordination between shared and individual goal-directed activities. Consistency across different therapists and centers will be maintained through clearly defined training activities and procedures, comprehensive protocol documentation shared among therapists and periodic inter-therapist meetings to review protocols and address potential challenges.

The therapist will oversee and adjust activities as needed to maintain the quality of the personalized intervention, documenting daily activities within a therapy diary.

### Sample size calculation

Using the GPower 3 software [79] with the “as in SPSS option”, we estimated that, with a mixed design between (group factor: active group vs. sham group) – within (variables within the subjects: Pre T0-, post T1-, follow-up T2, numerator DF = 2), a sample of 22 subjects per group allows detecting moderate to large effects (f (U) = 0.35), with a power of 0.80, setting a significance threshold of 0.05.

### Statistical analysis

Data analysis will be conducted using STATISTICA 8.0 (StatSoft Inc., Tulsa, Oklahoma).

Demographic and clinical variables of the two groups of patients will be inspected through descriptive statistics. Independent sample t test (two-tailed) and χ2 are used to assess the differences between the experimental and control training groups at baseline for continuous and categorical variables, respectively, thus allowing us to verify successful randomization. Normal distribution of outcome measurement data will be assessed using Kolmogorov-Smirnov tests. Should the data adhere to a normal distribution, repeated measures ANOVAs will be utilized to assess differences between treatment groups (active vs. sham) and across various time points (T0-T1-T2) for primary and secondary outcomes. Subsequent to identifying significant interactions, Duncan post hoc tests will be applied to address multiple pairwise comparisons effectively. Conversely, for non-normally distributed data, the Kruskal-Wallis H test or Friedman test will be employed. Additionally, an exploratory analysis will be conducted to explore potential sex and age-based differences in treatment response across primary and secondary outcomes. Statistical significance will be indicated by p < 0.05. As to what concerns missing data, a modified intention-to-treat analysis approach will be adopted, including in the analyses all the participants who had completed the pre- and post-treatment evaluation sessions, even if they had not completed all the training sessions. No imputation of missing data, however, will be used considering the limited sample size and observation points.

### Data safety and management

The project entails the processing and storage of pseudonymized data, which will be securely archived in a centralized repository situated on the servers of the study sponsor. Questionnaire scores and neuropsychological test results will not include any personal information regarding the participants, who will be distinguished solely by an alphanumeric code. Any records containing names or other personal identifiers, including informed consent forms, will be stored separately from the study records identified by code number. Access to the archives will be strictly controlled and granted solely through a rigorous authentication procedure, ensuring appropriate usage rights are maintained.

### Ethics and dissemination

Approval for this study was granted by the local ethics committee (Comitato Etico I.R.C.C.S. Eugenio Medea. Prot. N. 66/22-CE, approved on November 3, 2023; Comitato Etico Pavia, Prot. N. 0014336/23, approved on March 15, 2023; Comitato Etico Brescia, Prot. N. 5673, approved on January 23, 2023). Written informed consent will be obtained from the parents of every child participating. Minors as well will be asked to give their assent for participation. Each recruitment center will be responsible for collecting and securely storing the informed consent of every patient recruited within their facility. Subjects will be discontinued in the case of serious adverse events. Important protocol modifications will be reported to the Fondazione Regionale per la Ricerca Biomedica (FRRB) and clinicaltrials.gov, and approved by the Ethics Committees.

Patients and the public were not involved in the design of the study nor will be involved in its conduct, while patients’ associations will be involved in the dissemination plans of the results. The outcomes of this study will be shared with the involved physicians, referring practitioners, patients, and the broader medical community and disseminated through the official project’s website. Presentation of results is planned at academic and/or clinical conferences. Furthermore, the study aims to produce at least one peer-reviewed publication of quantitative findings in a reputable national or international medical journal. Reporting of this RCT will adhere to CONSORT guidelines [80] to ensure transparency and completeness in the presentation of results. The authors will adhere to principles of transparency and reproducibility in research by following the procedures presented in this study protocol and by sharing research material and anonymized dataset of this study in public repositories (https://osf.io/).

## Discussion

Functionality of the upper limbs plays a crucial role in a child’s ability to perform daily tasks and engage in various activities. However, children with CP often face challenges and exhibit frustration, particularly in two-handed tasks, compared to their typically developing peers [6]. Evidence-based recommendations advocate for personalized management programs, emphasizing collaborative goal-setting with children, parents, or caregivers, and incorporating physical therapy tailored to individual needs [8–11].

For years, the scientific community has debated the necessity of high-dose treatments. As argued in [81], our therapeutic actions—such as task selection and manipulation, practice methods, and feedback conditions—can significantly influence learning and skill generalization. The required dosage appears to vary depending on therapy content and objectives. Interventions that establish functional goals and incorporate actual goal-oriented practice tend to achieve goal attainment with lower doses compared to generalized upper limb motor training. In fact, while the notion of “more is better” is often presumed [82,83], some studies suggest otherwise, demonstrating that increased training does not necessarily yield greater improvements. Cope and Mohn-Johnsen’s review [84] on dose and practice intensity for children with CP concluded insufficient evidence to support high-dose intervention. Moreover, Novak’s review [85] underscores the importance of practicing goals for more than 30–40 hours to enhance general upper limb function (based on evidence in the unilateral population), and more than 14–25 hours to improve individual goals, requiring a combination of face-to-face therapy. Considering these findings, along with the cost and effort involved for children and their families, as well as the strain on the Health System, we have set the treatment dosage in our study at 30 hours. Although the inclusion of bilateral forms in our sample may introduce heterogeneity, setting a cutoff of 25% at the BBT allows us to select children with sufficiently disparate hand impairments to easily discern the most compromised hand for improved functionality in bimanual activities.

Beyond issues related to intervention dosage, there is an urgent need to identify rehabilitation approaches that yield significant clinical gains, not only in younger children, but also in more chronic conditions, such as older children and adolescents. This necessitates exploring new intervention forms that directly target brain plasticity. The rationale for our study stems from the growing body of evidence of NIBS potential therapeutic effects in adults with stroke and children with brain injuries [20–24]. Among tES techniques, tACS has garnered attention for modulating neuronal activity and behavior through weak alternating electrical currents delivered at specific frequencies [25]. Gamma oscillations, specifically, have been implicated in motor control, with aberrant gamma responses observed in children with CP [29,39,40]. Leveraging these findings, this study aims to assess the effectiveness of fronto-cerebellar gamma tACS integrated with HABIT-ILE in improving manual visuomotor control in pediatric CP patients. The fronto-cerebellar tACS montage targets specific cortical regions associated with motor control and integrates with the cerebellum, a key hub in motor learning networks [43,44]. Importantly, tailoring the stimulation protocol to each patient’s IGF aims to enhance intrinsic network dynamics and facilitate long-range connections between cortical regions [60].

In relation to outcome measures, this study has a number of strengths. Firstly, the chosen outcome measures exhibit both validity and reliability within our specific population of interest. Additionally, the incorporation of an ad-hoc created performance-based measure, along with an analysis of neurophysiological outcomes (cortical rhythms), holds promise for detecting potential treatment-related effects beyond the conventional clinical scales’ assessment. Furthermore, in-depth investigation of safety and tolerability of tACS with both psychophysiological and subjective reports will allow providing supportive evidence for the use of tACS, and of NIBS in general, in pediatric populations with motor disorders to boost the neurorestorative effects of rehabilitation.

## Supporting information

S1 Fig Schedule of enrolment, intervention and assessment at the defined time points. (PDF)

S1 Protocol Original protocol of the study. (PDF)

S1 Checklist SPIRIT checklist of the study protocol. (DOCX)
