# Supplementary material for: tACS-combined motor training for the rehabilitation of the upper limb in children and adolescents with cerebral palsy: A randomized, sham-controlled trial protocol
Source: PLoS One. 2025 Sep 3;20(9):e0331360. doi: 10.1371/journal.pone.0331360 (PMC12407442; doi:10.1371/journal.pone.0331360)
Supplement: S1 Fig — (PDF) [file pone.0331360.s001.pdf]

		STUDY PERIOD				
		**Enrolment**	**Allocation**	**Intervention**	**Post-intervention**	**Follow-up**
**TIMEPOINT**		T0	T0		T1	T2
**ENROLMENT:**						
	**Eligibility screen**	X				
	**Informed consent**	X				
	**Allocation**		X			
**INTERVENTION**:						
	**Active tACS + HABIT-ILE**			X		
	**Sham tACS + HABIT-ILE**			X		
**ASSESSMENTS:**						
Training feasibility	*Number of dropouts*				X	
	Number of sessions completed per child				X	
Training acceptability	*Ad-hoc questionnaire child- and parent-compiled*				X	
Training safety and tolerability	*Measure of capillary saturation of O2*			X		
	Heart rate measure			X		
	Skin irritations			X		
	Ad-hoc self-perception questionnaire for stimulation-induced sensations			X		
**PRIMARY OUTCOMES:**						
**Hand spontaneous use**	*Assisting Hand Assessment*		X		X	X
	Box and Block test		X		X	X
**Visuomotor control**	*Performance in the computer-based Visuomotor task*		X		X	X
**SECONDARY OUTCOMES:**						
**Motor functions**	*Children's Hand-use Experience Questionnaire*		X		X	X
	Canadian Occupational Performance Measure		X		X	X
	Melbourne Assessment-2		X		X	X
	Gross Motor Function Measure		X		X	X
**Functional adaptation**	*Vineland Adaptive Behavior Scale Version 2*		X			X
**Quality of life**	*Pediatric Quality of Life Inventory*		X			X
