# Supplementary material for: tACS-combined motor training for the rehabilitation of the upper limb in children and adolescents with cerebral palsy: A randomized, sham-controlled trial protocol
Source: PLoS One. 2025 Sep 3;20(9):e0331360. doi: 10.1371/journal.pone.0331360 (PMC12407442; doi:10.1371/journal.pone.0331360)
Supplement: S1 Protocol — (PDF) [file pone.0331360.s002.pdf]

Polo di Bosisio Parini
Via don Luigi Monza, 20
23842 Bosisio Parini (LC)
tel. +39 031 877111
fax. +39 031 877223
polo.bosisio@lanostrafamiglia.it
direzione.bosisio@pec.emedea.it
www.emedea.it

PROTOCOLLO DI STUDIO

TITOLO DELLO STUDIO:	**Bottom-up and tOp-down neuromOdulation of motor plaSTicity in cerebral palsy - BOOST**
NUMERO DI VERSIONE DEL PROTOCOLLO:	Id. 944 - Versione n. 0 del 26.09.2022<br>Codice FRRB: 3438840
FINANZIAMENTO:	Fondazione Regionale per la Ricerca Biomedica FRRB - BANDO "UNMET MEDICAL NEEDS"
PROMOTORE:	IRCCS Eugenio Medea – sezione scientifica dell'Associazione "La Nostra Famiglia"
CENTRO COORDINATORE:	IRCCS Eugenio Medea – sezione scientifica dell'Associazione "La Nostra Famiglia" – Via Don Luigi Monza, 20, 23842 Bosisio Parini (LC)
SPERIMENTATORI COINVOLTI:	IRCCS EUGENIO MEDEA<br>Dr.ssa Alessandra Finisguerra - Ricercatrice, Psicologa;<br>Prof. Cosimo Urgesi - Professore Associato di Psicologia dello Sviluppo e dell'Educazione dell'Università di Udine e Ricercatore;<br>Dr.ssa Viola Oldrati - Collaboratrice alla Ricerca, Psicologa.<br><br>FONDAZIONE MONDINO IRCCS - ISTITUTO NEUROLOGICO NAZIONALE<br>Prof. Renato Borgatti - Professore Ordinario di Neuropsichiatria dell'Infanzia e dell'Adolescenza, Neuropsichiatra dell'Università di Pavia;<br>Prof.ssa Simona Orcesi - Professoressa Associata di Neuropsichiatria dell'Infanzia e dell'Adolescenza, Neuropsichiatra.<br><br>UNIVERSITÀ DEGLI STUDI D BERGAMO<br>Prof.ssa Zaira Cattaneo - Professoressa Associata di Psicobiologia e Psicologia Fisiologica, Psicologa.<br><br>ASST DEGLI SPEDALI CIVILI DI BRESCIA<br>Prof.ssa Elisa Fazzi - Professoressa Ordinaria di Neuropsichiatria dell'Infanzia e dell'Adolescenza, Neuropsichiatra.

	Prof.ssa Jessica Galli - Professoressa Associata di Neuropsichiatria dell'Infanzia e dell'Adolescenza, Neuropsichiatra.<br>Dr. Andrea Rossi - Neuropsichiatra
CENTRI COINVOLTI:	1. FONDAZIONE MONDINO IRCCS - ISTITUTO NEUROLOGICO NAZIONALE - Via Mondino, 2, 27100 Pavia (PV) – Prof. Renato Borgatti<br>2. UNIVERSITÀ DEGLI STUDI D BERGAMO - via Salvecchio 19, 24129 Bergamo (BG) - Prof.ssa Zaira Cattaneo<br>3. ASST DEGLI SPEDALI CIVILI DI BRESCIA - Piazzale Spedali Civili, 1 25123 (BS) – Prof.ssa Elisa Fazzi

Informazioni di Contatto

NOME CONTATTO PROMOTORE:	Dr.ssa Alessandra Finisguerra<br>Tel. 031/877946<br>Email: alessandra.finisguerra@lanostrafamiglia.it
NOME CONTATTO PER LA DISPOSITIVO-VIGILANZA:	Ing. Paola Grigioni

APPROVAZIONE DEL PROTOCOLLO

Gli Sperimentatori:

- approvano il presente Protocollo;
- dichiarano che lo studio verrà condotto in conformità alle Good Clinical Practice secondo quanto riportato nel presente protocollo.

Data	Nome dello Sperimentatore Locale	Firma
______________	Dr. Alessandra Finisguerra	
______________	Prof. Renato Borgatti	
______________	Prof.ssa Elisa Fazzi	
______________	Prof.ssa Zaira Cattaneo	

Indice

Background e razionale 5
Obiettivi dello studio 6
Considerazioni etiche 8
The studies will be carried out in compliance with the protocol, Good Clinical Practice guidelines, the ethical principles that have their origin in the Declaration of Helsinki and the applicable regulatory requirements. 8
Modalità per l'ottenimento del consenso informato 8
Popolazione vulnerabile e trattamenti in circostanze di emergenza 8
Prove precliniche e esperienza clinica precedente 9
Informazione relative alla indagine clinica 10
Piano di monitoraggio dell'indagine clinica 20
Assicurazione della qualità, procedure di controllo, gestione dei dati e conservazione della documentazione 22
Deviazioni al piano di valutazione clinica 22
Eventi avversi 22
Emendamenti al piano di valutazione clinica 24
Termine anticipato e sospensione della valutazione clinica 24
Statistica 24
Politica di pubblicazione dei dati 25
Bibliografia 25

Background e razionale

Cerebral Palsy (CP) refers to a group of nonprogressive permanent disorders causing movement and postural impairments, originating from insult to the immature brain and having, with accompanying neurological and neuropsychological impairments, important impact on patients and families. Different protocols of neuromotor rehabilitation have been proposed to alleviate the impact of CP. Goal-directed occupational therapies in which the child is actively engaged in practicing a task to achieve a desired goal are associated with greater improvements in motor and self-care outcomes, with respect to movement-based interventions (Jackman et al., 2020). These protocols seem to provide larger gains with more intensive doses (at least 30hr of practice), particularly for upper limb motor rehabilitation (Jackman et al., 2020), and at earlier stage from the insult (i.e., very young children), with a decrease of effectiveness as children grow and brain plasticity decreases (Cioni et al., 2011). Non Invasive Brain Stimulation (NIBS) may adjuvate neurorehabilitation (Finisguerra et al., 2019) by promoting neuroplasticity also in chronic conditions and by modulating neural activity and recovery after brain injury in a noninvasive way (O'Leary et al., 2021). Recent studies have provided preliminary evidence on the safety, tolerability, and efficacy of combining NIBS with functional training to help minimize motor impairments in children with CP. NIBS may promote the activation of the primary motor cortex and lead to positive effects on both immediate and long-term motor functions (Fleming et al., 2018). However, the overall effect size of the studies has be so far limited, with large response variability according to patients' demographic (e.g., age), clinical (e.g., stroke type and side of lesion), and neurophysiological motor profile (e.g., corticospinal excitability and intracortical inhibition) factors (Kuo et al., 2022). There is thus an unmet need for developing new NIBS protocols tailored to the individual pattern of neurofunctional organization and that may provide entries for targeting the motor system through non-lesioned structures. A challenging possibility is to promote motor-recovery in patients with CP by acting on spared networks that are involved in the tuning of neural activity in the motor system. More specifically, it is possible to promote motor plasticity in a bottom-up vein by stimulating the vagus nerve (VN) and in a top-down vein by acting on the fronto-cerebellar circuit. Activation of the VN propagates to subcortical, and cortical brain structures, enhancing brain GABA (Marrosu et al., 2003) and Noradrenaline levels, which play a pivotal role in brain plasticity. GABA-A receptor–mediated neurotransmission is particularly important to promote practice-dependent plasticity after brain injury and its alterations is considered an important patho-physiologic mechanism of motor dysfunction in CP (Park et al., 2013). Accordingly, studies on animal models have demonstrated that invasive VN Stimulation (VNS) combined with motor practice promotes cortical reorganization, leading to greater improvement in forelimb motor recovery and increased synaptic motor connectivity as compared to motor practice alone (Meyers et al., 2018). In humans, a recent study (Dawson et al., 2021) has documented the beneficial effects (persisting after 90 days) of invasive VNS paired with physical rehabilitation, as compared to rehabilitation alone, in adults with moderate-to-severe arm impairment after chronic ischemic stroke. Unfortunately, invasive VNS necessitates a costly surgical procedure. Non-invasive

activation of the VN can be achieved by delivering electrical pulses to the sensory afferent fibers of the auricular, thick-myelinated, branch of the vagus nerve in the outer ear (Farmer et al., 2021), with transcutaneous VNS (tVNS). TVNS seems to engage the same neural pathways of invasive VNS methods (Assenza et al., 2017) and may provide a novel, bottom-up NIBS method to promote cortical plasticity.
A growing number of studies suggests that an effective way for top-down modulation of cortical activity is the use of transcranial alternating current stimulation (tACS) (Wessel et al., 2022), which affects spontaneous brain oscillations via low-intensity alternating currents applied on the scalp surface. Frequency of stimulation can be tuned (i.e., entrained) to the natural frequency of the underlying oscillations. The cerebellum represents a promising target candidate for this stimulation approach as it plays a crucial role in adapting and fine-tuning of movements (Manto et al., 2012) and has been proposed to contribute to the synchronization of activity within and between distributed brain regions. Indeed, cerebellar tACS may influence long-range fronto-cerebellar connections, inducing online and after-effect improvements of motor performance (Naro et al., 2016, 2017).

Obiettivi dello studio

This project aims to optimize and trial novel NIBS protocols tailored to the individual clinical profile of CP patients, namely tVNS and tACS, which provide, respectively, a bottom-up and a top-down pathway to stimulation of neural plasticity. We first test the neurophysiological mechanisms of action in inducing neuroplastic changes of motor cortex in young adults with typical development, by using state-of-the-art neurophysiologic and electroencephalographic measures of cortico-spinal excitability and intracortical inhibition. We hypothesized that active tVNS and tACS but not sham tVNS or tACS will induce a change in corticospinal measures of excitation and inhibition, on cortical plasticity and behavioral measures of motor abilities in healty adults. Then, we translate this knowledge to a two-arm, randomized, double-blind, sham-controlled trial of the efficacy of tACS and tVNS combined with a gold-standard motor rehabilitation intensive training in 6-17 yo CP patients with mild-to-moderate upper-limb deficits. We hypothesized that tACS and tVNS may potentiate the effects of functional training by promoting brain plasticity and boosting motor learning. More specifically, the combined active NIBS + intensive training treatment we hypothesized should induce to a greater improvement in the tested functions (see the session *Variabili da misurare*) with respect to the sham NIBS + intensive training treatment. In this last case, an improvement in the post treatment session is anyway expected. tVNS will be performed by using a CE marked class IIa tVNS device (tVNS®*E*; tVNS technologies GmbH, Erlangen, Germany). tVNS®*E* is intended for all patients of the age of 6 or above; for patients suffering from a variety of disorders including stroke patients (see the tVNS_E_IFU_EN InvestigatorsBrochure). tACS will be performed by using the Starstim 8 System (Neuroelectrics, Barcelona, Spain). This is intended for inducing cortical neuromodulation for the treatment of neurological disorders (see the Starstim_EC_EN InvestigatorsBrochure).

Valutazione del rapporto beneficio/rischio potenziale per la popolazione

Healthy participants (WP1 & WP2):
There will be no direct benefits for the healthy adults undergoing the tVNS or the tACS experiments. However, they will be informed that by participating to these studies they will promote the improvement of the treatments of children and adolescents with CP. Potential risks /unwanted effects will be related to fatigability of participants. For these reasons, to minimize fatigability and to reduce the number of TMS pulses within the same day, the experimental procedure will be subdivided into two sessions, in separate days. Risks can be associated to the presence of contraindication to NIBS. In order to avoid any potential side effects, the samples of healthy adult participants will be screened for possible contraindication prior to the inclusion into the study. Medication intake and medical condition will also be taken into account.

CP Patients (WP3 & WP4):
Each group of CP patients participating to this project will benefit from the intensive bimanual treatment. CP patients that will be assigned to the active stimulation (tVNS or tACS) group could exhibit a greater improvement in the tested outcomes with respect to the sham groups. Potential risks can be linked to the Treatment tolerance. tVNS and tACS are usually well tolerated and safe in both the pediatric (Krishnan et al., 2015) and adult populations (Antal et al., 2017; O'Leary et al., 2021). In a study examining tolerability in children with CP (Alon et al., 1998), there were no reports of seizures, episodes of nausea or vomiting, or sleep disruption by any of the participating children or their parents; all children tolerated the active stimulation procedure well. In any case, participants will be asked to report unwanted effects and vital parameters (oxygen saturation, heart rate) will be measured. To reduce potential effects on the heart during tVNS, the electrode will be applied to the left ear, as the vagal fibers directed to the heart are coming from the right side. The stimulation intensity will be set based on the perceptual threshold of the participants, corresponding to the perceived intensity and below the perception of pain to minimize discomfort. In the tACS studies, an electrical current strength of 1 mA (lower than the dose applied in WP2) will be delivered in order to compensate for the thinner skull and lower resistance of younger participants (Antal et al., 2017). Moreover, an intra-cephalic montage will be tested, whereas cardiac safety issues have been posed in relation to extra-cephalic montages. In both cases, either for tVNS and tACS studies participants will be thoroughly screened against the defined exclusion criteria to minimize the risks associated with the application of the stimulation. Medication intake will also be considered.

No participants will receive financial compensation for participation in the study.

Ritiro dei soggetti e modifiche dell'intervento
Participants and their parents/guardians in the case of minors will be informed about the procedures of the studies and they will be asked to sign the written informed consent. All doubts presented by the participant or parents/guardians will be resolved, and all of them will be able to revoke their consent at any time for any possible reasons if they wish to do so without any consequences. The experimenters could evaluate to withdraw the participant from the study if he/she will show discomfort (VAS discomfort score > 9 in more 50% of unwanted sensations) or if her/him medical

conditions/drugs therapy /inclusion criteria to NIBS will change throughout the experimental sessions or for other urgent medical reasons. Participants will be randomly allocated to the real or the sham tVNS group and patients will be randomly allocated to the real or the sham tVNS or tACS -combined motor training arm, and then to real- or sham-condition. We did not consider any change in patients' allocation.

Conclusione anticipata o sospensione dello studio

The coordinator may interrupt the study at any time and promptly notify the ethics committees and all partners. Patients will be informed as soon as possible and they will continue to be followed up according to normal clinical practice.

Definizione di conclusione dello studio

For healthy adult participants involved in WP1 or WP2 the involvement will finish after the end of the second session. For CP patients involved in WP3 or WP4 the involvement will finish after the follow up sessions (t2), three months after the conclusion of the treatment. The project will end after the follow-up session of the last recruited patients or after three years since its beginning.

Considerazioni etiche

The studies will be carried out in compliance with the protocol, Good Clinical Practice guidelines, the ethical principles that have their origin in the Declaration of Helsinki and the applicable regulatory requirements.

Modalità per l'ottenimento del consenso informato

Prior starting each study, all participants and their parents/guardians for the studies involving minors will receive an oral and a written description of the protocols, the potentials risks and benefits of the study in which they will be involved (see the *Informativa e consenso* modules). Three different versions will be used, for adults (older than 18), adolescents (12-17 yo) and children (6-11 yo), to ensure the full understanding of the information for each participant. Thus, written informed consent will be obtained for either all participants or, if their capacity to consent is absent and for minors, their parents/guardians. A copy of the consent will be given to the participant; the other will be stored by the experimenter. All personal information about participants will be prohibited from other use and stored in specific cabinets in order to protect confidentiality before, during, and after the study (see the *Informativa e consenso dati* module).

Popolazione vulnerabile e trattamenti in circostanze di emergenza

Although it is not excluded the possibility that some of the healthy participants taking part in Wp1 and Wp2 are students attending some courses held by the head of the study during the time frame of the research or in the near future, participation will take place through voluntary participation.

Children and adolescents aged 6 -17 yo diagnosed with CP will be involved in the studies. Patients with CP will have unilateral or bilateral upper limb impairments, resulting from perinatal stroke. Their diagnosis will be confirmed according to Surveillance of Cerebral Palsy (SCPE) criteria (Sadowska et al., 2020). For each study, a physician will be present in the structure during the experiments and it will be directly consulted by the researcher when an adverse event occurs. tVNS, tACS or TMS stimulation might be somewhat uncomfortable and can incidentally cause a mild headache. The headache can effectively be treated by light analgesics (i.e., 500 mg paracetamol). In case of adverse events participant will end the study and he/she will be followed until the events have abated, or until a stable situation has been reached. Additional tests or medical procedures will be discussed and proposed with the general physician or a medical specialist.

Prove precliniche e esperienza clinica precedente

The promotor MEDEA, and the other clinical centres (Mondino and ASST Spedali Civili) have long-lasting experience in the evaluation and treatment of patients with CP. The investigators at Medea and University of Bergamo UNIBG are expert in the use of these techniques, in healthy and in clinical populations. A previous study (Dawson et al., 2021) documented the beneficial effects (persisting after 90 days) of invasive VNS paired with physical rehabilitation, as compared to rehabilitation alone, in adults with moderate-to-severe arm impairment after chronic ischemic stroke. Unfortunately, invasive VNS necessitates a costly surgical procedure. Non-invasive tVNS approaches have been developed as a less expensive, patient friendly and rapidly deployable alternative. Different devices for noninvasive vagus nerve stimulation are nowadays commercially available. Among these device, the VNS- E is intended for all patients of the age of 6 or above; for patients suffering from a variety of disorders including stroke patients. While the potentialities of invasive VNS to promote motor plasticity and recovery has been documented in animal models (Meyers et al., 2018) and recently trialed in adult patients with acquired brain damage (Dawson et al., 2021), to our best knowledge no study has so far tested the feasibility and efficacy of tVNS combined with neuromotor training in CP. TVNS has been largely applied in pediatric populations to treat other disorders, mainly epilepsy, and is considered safe and tolerable (O'Leary et al., 2021).
With respect to tES, the study by (Alon et al., 1998) assessed the effects of tACS in 7 children (age range 2.5, 7.5 yo) with CP. No adverse reactions occurred. The effects of tDCS in CP were also tested (Duarte et al., 2014; Grecco et al., 2014). With respect to the effects of tVNS on corticospinal excitability or cortico-cortical inhibition, to date, only one study (Capone et al., 2015) directly assessed the effects of tVNS on cortical excitability in 10 healthy participants, reporting an increased short-interval intracortical inhibition (SICI), which is known to mirror GABA-A activity. The effects of tVNS on other indices of excitatory/inhibitory balance of cortical activity and its effects on cortical plasticity remain mostly unclear. Available evidence shows that gamma entrainment by cerebellar tACS can improve motor performances and modulate cerebellar-cerebral interactions (Naro et al., 2016). Furthermore, the gamma oscillations are thought to play an important role in the binding among cortical areas involved in complex motor tasks (Ulloa, 2021).

Overall, the experimental plan of this project is also supported by preliminary findings of the partners that have provided indications about the most effective cerebellar tACS protocol in terms of stimulation focality and previous experience in tVNS protocols with minor patients and healthy participants with tVNS. With respect to tACS protocol, the fronto-cerebellar electrode montage (i.e., active electrode over the right cerebellar hemisphere combined with a reference electrode over left frontal F3) has been demonstrated to be safe, feasible to be applied in pediatric clinical populations. The feasibility of tVNS protocols in minor patients and healthy adults emerged from two ongoing study of the Coordinator-MEDEA (Prot. N. 22/19 - Comitato Etico Regionale Unico, Friuli Venezia Giulia, Italy, Prot. N. 23460; Sper 82) using tVNS to improve noradrenaline and GABA related functions assessed by single pulse SP and paired pulse PP TMS protocols. In these studies, tolerability of tVNS was assessed by recording pre vs. post tVNS heart rate (HR) and by administering a self-report questionnaire about emotional states and side-effect sensations experienced. No major adverse effects, no changes in bradycardia were recorded. Overall, preliminary findings suggest positive effects of the stimulation on the investigated cognitive functions, and no adverse effects due to the tVNS, neither in patients nor in controls. Despite the small numerosity of the patient sample, which prevented a rigorous statistical approach to the data, these preliminary observations confirm the safety, the tolerability and feasibility of the treatment with pediatric patients and of the stimulation in healthy adults.

Informazione relative alla indagine clinica

Descrizione dello studio clinico

This is a multicentre controlled randomized project that will be carried out in Lombardia region. The project is articulated in 4 work packages. In a first stage of the project it aims at ***investigating state dependent effects of tVNS on cortical excitability and plasticity (WP1)*** and testing the effects of ***EEG-guided transcranial alternating current stimulation (tACS) of the cerebellum (WP2) in healthy adults*.** MEDEA and UNIBG units will model and test in healthy individuals the most effective stimulation parameters to promote motor performance and cortical plasticity. This would allow us to identify the NIBS induced markers of plasticity within the motor system and the relative interindividual differences. In the second phase of the project (WP3 and WP4) children with CP will be recruited, treated and evaluated at the MEDEA, MONDINO and SPEDALI CIVILI recruitment centers. In particular, two randomized sham controlled trials will performed to test the effects of ***tVNS-combined motor training (wp3)*** and ***tACS-combined motor training (wp4) for the rehabilitation of the upper limb in children and young people with cerebral palsy.***

WP1: Investigating state dependent effects of tVNS on cortical excitability and plasticity.

WP1 will be coordinated by Partner 1-MEDEA (Dr. Finisguerra and Prof. Urgesi) in collaboration with Partner2-UNIBG. Two studies are comprised in this Wp. For each study, a group of 44 adult participants (18-25 yo, 22 women and 22 men) will be recruited at the post-graduate courses hosted by MEDEA via announcements posted on the classroom board. In both studies, a within-subject placebo-controlled single blind design will be adopted. Each participant will undergo a 2-session

experiment, during which active or control (sham) tVNS will be applied during the execution, with the dominant and nondominant hand) of a computerized visuomotor task (see WP2). In both studies, tVNS will be performed by using a CE marked class IIa tVNS device (tVNS®*E*; tVNS technologies GmbH, Erlangen, Germany).

Study 1. Following tVNS, different measures of cortical excitability and inhibition will be taken. In particular, following standard procedures, the resting motor threshold (rMT), the Input/Output recruitment curve (RC), and the cortical silent period (cSP) will be recorded by applying single-pulse TMS protocols. Paired-pulse TMS protocols will be used to measure the Short (SICI) and Long interval intracortical inhibition (LICI).

Study 2. For each session, after 60 min of tVNS, we will apply the Paired Associative Stimulation (PAS) protocol, which has been proposed as an assay of central nervous system plasticity in patients with chronic stroke.

WP2: EEG-guided transcranial alternating current stimulation (tACS) of the cerebellum

WP2 will be led by Partner 2-UNIBG (coordinated by Prof. Z. Cattaneo), which will carry on the recruitment of participants, data collection and analysis and dissemination of results, in collaboration with Coordinator-MEDEA. Forty-four healthy adult participants (18-25 yo; 22 men and 22 women) will be recruited at the UNIBG via announcements posted on the board at University. Data collection will be carried out in a structure under medical supervision. The study of WP2 consists of two sessions. In the first session (part 1), 5 min eyes-open resting state cortical activity will be recorded. Then, participants will perform a computerized visuomotor task during the EEG recording in a one-on-one, distract-free testing environment. Participants will be seated in front of a table facing a computer with their dominant or nondominant hand holding a joystick/mouse. The joystick/mouse will be used to collect the digitized data of the participants' hand movements. Visual feedback of the joystick/mouse movements will be displayed and will be provided to the participants in real-time on a computer monitor. Participants will be asked to move a cursor using the joystick/mouse as straight and as fast as possible between starting positions and target positions displayed on the computer screen. The speed and accuracy of participants pointing movement will be collected and analyzed. This task has been previously used to study visuomotor adaptation in children (Lee & Bo, 2021) and allows recording accuracy and reaction times to provide a more sensitive measure of motor performance. The EEG recordings will be analyzed to detect the individual prominent spectral peak within the gamma bandwidth – or individual gamma frequency (IGF) – emerged in the prefrontal electrodes. The IGF will be selected as the target frequency to be-delivered by means of tACS. Participants will then perform again the computerized visuomotor task (part 2) during the administration of the cerebellar tACS. Immediately after the end of the stimulation, indices of motor excitability and cortical inhibition will be assessed following the same procedure applied in WP1. The sham control condition will be delivered in a separate session.

WP3: tVNS-combined motor training for the rehabilitation of the upper limb in children and young people with cerebral palsy: a randomized, sham-controlled trial

WP3 will be led by Coordinator-Medea (led by Dr. Finisguerra and Prof. Urgesi), which will coordinate protocol design, centralized data analysis, and dissemination. Participants will be recruited, treated, and evaluated at the MEDEA, MONDINO and SPEDALI CIVILI recruitment centres. The multicenter study applies a randomized, pre-/post-test and sham-controlled trial design. Once allocated to the tVNS- rather than tACS-combined motor training arm, patients will be allocated to real- or sham-tVNS, using a stratified permuted block randomization procedure. Children and adolescents aged 6 - 17 yo diagnosed with CP will be recruited from patients assisted in the recruitment centres. Before the start of the rehabilitation program, in a separate experimental session, patients will perform with the less affected hand the computerized visuomotor task (tested in WP1-WP2) during the delivery of either active or sham tVNS, according to group assignment, to examine the acute effects of tVNS on motor performance (one-shot experiment). A between-subject design, constrained to group assignment, will be used to avoid compromising blinding. The rehabilitation treatment will be carried out for 5 consecutive days, for 3h/day for 2 weeks, for an overall time interval of 30 hours and consists of a bimanual training in an ecological and highly motivating environment. Patients will be treated in pairs, with a matching of motor deficit severity, IQ, and age. The choice of the type of activities to be practiced in each session (e.g., learning to roll out the dough for a pizza party, to weave a tale for an exhibition, or peel apples for an apple cake) will be left to patients, boosting the motivational aspect of the training. Children will be assessed before the start of treatment (T0), immediately after the end of intensive treatment combined with tVNS delivery (T1) and three months after the end (T2). Pre- and post-treatment evaluation sessions are administered to both the experimental and the control groups (i.e., active vs. sham tVNS). Real and sham tVNS will be applied as described in WP1. The tVNS, active or sham, will be applied starting 15 minutes before the training and for the first hour of its course.

WP4: tACS-combined motor training for the rehabilitation of the upper limb in children and young people with cerebral palsy: a randomized, sham-controlled trial

WP4 will be led by MONDINO (coordinated by Prof. R. Borgatti), in collaboration with UNIBG (coordinated by Prof. Z. Cattaneo), which will be in charge of protocol design, centralized data analysis, and dissemination. Participants will be recruited, treated and evaluated at the MEDEA, MONDINO and SPEDALI CIVILI recruitment centres. The multicenter study applies a randomized, pre-/post-test and sham-controlled trial design. Once allocated to the tACS- rather than tVNS-combined motor training arm, patients with CP will be allocated to real- or sham-tACS, using a stratified permuted block randomization procedure. Before the start of the rehabilitation treatment, in a separate experimental session, patients will participate in a one-shot experiment, aimed at identifying the IGF and at examining the online effects of tACS on the computerized visuomotor task tested in WP2. Patients will perform, with the less affected hand, the task during the EEG recording, according to the technical procedure applied in WP2. Subsequently, the EEG recordings will be

analyzed to detect the IGF emerged in the prefrontal electrodes. The IGF will be selected as the target frequency to be-delivered by means of tACS. Participants will perform once again the computerized task during the delivery of either active or sham tACS, according to group assignment. The further step consists in the tACS-combined rehabilitation program. Thus, patients will be assessed before the start of treatment (T0), immediately after the end of intensive treatment combined with tACS delivery (T1) and three months after the end (T2). The tACS will be applied for 20 minutes, following the same procedures of Wp2, but the intensity of the stimulation will be halved to 1 mA.

Obiettivi primari e secondari

The primary aim of the project is to optimize and trial the integration of tVNS and tACS protocols with an intensive bimanual training in an ecological and highly motivating environment in order to boost the rehabilitative outcome of patients with CP.

Throughout the four WPs, in a first phase with healthy adults the project aims at assessing:

i) the state dependent effects of tVNS on cortical excitability and cortical inhibition (Study 1, Wp1) and ii) on cortical plasticity (Study 2, Wp2); iii) the effects of gamma tACS with a fronto-cerebellar montage, on the execution of a computerized visuomotor task and iv) whether cerebellar tACS might increase cortical plasticity in the motor cortex.
In a following phase with children and adolescents with CP, it aims at i) testing the feasibility and efficacy of a tVNS-combined rehabilitation treatment of the affected upper limb in CP and ii) the acute effects of tVNS on the behavioral execution of a visuomotor task; iii) testing the feasibility and efficacy of a tACS-combined rehabilitation treatment of the affected upper limb in patients with CP and iv) the acute tACS effects on the behavioral execution of the computerized visuomotor task.

Endpoint

Wp1.

Statistically significant ($p< 0.05$) differences in the active vs the sham tVNS condition in measures of corticospinal excitation, cortical inhibition and plasticity; non statistically significant ($p< 0.05$) differences between active and sham tVNS in the tolerability outcomes

Wp2

Statistically significant ($p< 0.05$) differences in the active vs the sham tACS condition in brain oscillations, in measure of corticospinal excitation, and cortical inhibition and in behavioral performance in the visuomotor tasks; non statistically significant ($p< 0.05$) differences between active and sham tVNS in the tolerability outcomes

Wp3 and Wp4

Statistically significant ($p< 0.05$) differences from t0 to t1 and/or to t2 in the active vs the sham NIBS stimulation paired with the intensive bimanual training in the clinical measures (see *Variabili da misurare*); non statistically significant ($p< 0.05$) differences between active and sham NIBS in the tolerability outcomes

Variabili da misurare

Wp1:

Efficacy outcome:

- Resting motor threshold (rMT)
- Input/Output recruitment curve (RC)
- cortical silent period (cSP)
- Short (SICI) and Long interval intracortical inhibition (LICI)
- Pre-Post PAS baseline MEPs
- behavioral performance in the visuomotor task (speed, accuracy of pointing movements)

-

Safety and tolerability outcome:

- SPO2
- HR
- Adults version of visual analogue scales for discomfortable sensations

Wp2:

Efficacy outcome:

- individual gamma frequency (IGF) during the visuomotor task
- Resting motor threshold (rMT)
- Input/Output recruitment curve (RC)
- cortical silent period (cSP)
- Short (SICI) and Long interval intracortical inhibition (LICI)
- behavioral performance in the visuomotor task (speed, accuracy of pointing movements)

Safety and tolerability outcome:

- EEG alterations
- Adults version of visual analogue scales for discomfort sensations

Either for Wp1 or Wp2, as Efficacy outcome, the electromyography EMG signal will be recorded through a Biopac MP-36 Systems. The peak to peak amplitude and the latency of the MEP recorded from the FDI muscle will be analysed. The epoch analyses of the signal will be performed on a temporal windows of 50 ms. The safety and tolerability measures will be reported on participants file data “Scheda raccolta dati dello studio”. See the “statistica” session for a detailed description of the analyses.

Wp3 and Wp4.

Efficacy outcome:

- Canadian Occupational Performance Measure (COPM);
- Children’s Hand Use Experience Questionnaire (CHEQ);
- Box and Block Test (BBT);

- Melbourne assessment scale of upper limb motor functions (MUUL);
- Assisting Hand Assessment (AHA);
- Gross Motor Function Measure (GMFM);
- Vineland Adaptive Behavior Scales (VABS);
- behavioral performance in the visuomotor task (speed, accuracy of pointing movements)

Safety and tolerability outcome:
- SPO2
- HR
- EEG alterations
- Child/Adolescent version of visual analogue scales for discomfort sensations

The scores of participants in the clinical scales, the safety and tolerability measures will be reported on participants file data "Case Report Form". See the "statistica" session for a detailed description of the analyses.

Errori sistematici - bias

To prevent bias associated with *patients' and investigators' expectation*, different blinding procedures will be applied. Wp1 and Wp2: a single blind design will be adopted, in which the participant, but not the experimenter, will be blind to the real vs sham tVNS/tACS condition. Wp3 and Wp4: the staff who will carry out the clinical, instrumental, and training evaluation will be blind to group assignment. Instead, the coordinator of the study, the staff who will apply the tVNS and analyze the data will not be blinded to group allocation. To avoid a *selection bias* of patients assigned to the active or the sham intervention group could lead to unevenly distribution of prognostic factors between the experimental and the control group, stratified sampling on the basis of age and clinical severity will be used. Once divided, participants within each subgroup will be randomly assigned to the intervention treatment. Moreover, effects of *interindividual variability* will be considered as follows. Wp1 and Wp3: Effects due to possible confounding variables contributing to inter-individual differences in tVNS responses (individual baseline level of vagal activation and hormones, age, sex; (Farmer et al., 2021; Koenig et al., 2017; Warren et al., 2019). Although the use of a within-subject placebo-controlled single blind design in Wp1 should reduce the impact of interindividual differences, the baseline vagal activation as indexed for example by resting-state heart rate variability (HRV) will be recorded. Moreover, given possible age and gender differences in baseline vagal activation, an equal number of male and female participants will be recruited and stratified by age. All these factors will be considered in the analysis to examine their potential influence on the effect of tVNS. Medication intake and medical condition will also be taken into account. Wp2 and Wp4: Response variability according to gender of participants: the factor gender will be considered in the analysis in order to examine its potential influence on the effect of tACS. Moreover, in Wp2 a within subject placebo-controlled single blind design will be adopted. Wp3 and Wp4: Variability according to individual preferences toward bimanual activity. To avoid this bias, each participant will choose the recreational play activities to perform in the motor rehabilitation program. In addition to maintaining

a highly motivating environment, this will help avoiding cultural gender biases in the identification of those activities. Furthermore, on a methodological level, this choice will help to rule out that common preconceptions on what may engage female and male participants differently would influence training feasibility and efficacy. Drop out rate higher the 30% of the estimated sample size. This would hamper the possibility to reach the desired power and to obtain follow up measures.

Selezione dei pazienti

In order to be eligible to participate in WP1 or WP2, healthy participants must meet all of the following criteria:

- Between 18-25 years of age;
- Normal or corrected-to-normal vision;
- Willingness and ability to give written informed consent and willingness and ability to understand the nature and content, to participate and to comply with the study requirements.

A potential participant who meets any of the following criteria will be excluded from participation in this study:

- Epilepsy or familiarity with epilepsy;
- Migraine;
- ferromagnetic metal parts in the head (except for a dental wire);
- Implanted cardiac pacemaker or neurostimulator;
- Pregnancy.

Participants' answers to the following questions (*in Italian*) will be carefully evaluated:

- Soffro o ho sofferto di crisi epilettiche o assenze *
- Qualcuno nella mia famiglia ha avuto crisi epilettiche o assenze
  Se sì, indicare il grado di parentela
- Soffro di emicrania*
- Qualcuno nella mia famiglia soffre di emicrania
  Se sì, indicare il grado di parentela
- Sono portatore di pompa di infusione per insulina o altri farmaci
- Sono portatore di pace maker cardiaco o altri tipi di catetere cardiaci*
- Sono portatore di protesi del cristallino
- Sono portatore di clip metalliche e /o craniche o comunque del metallo in qualunque parte della testa eccetto la bocca*
- Sono portatore di neurostimolatori, elettrodi, impianti nel cervello o subdurali*
- Soffro di una malattia cardiaca
- Se si quale?____
- Soffro di qualche disturbo medico, psichiatrico, o neurologico.
- Se si quali?____
- Soffro di malattie cutanee?
- Se si quali?____

- Sto assumendo farmaci/ ho di recente interrotto l'assunzione prolungata di alcuni farmaci?

Se si quali/da quanto tempo è stata interrotta l'assunzione?____

- Abitualmente faccio uso di sostanze stupefacenti o abuso di sostanze alcoliche
- Ho abusato di sostanze come alcol o droghe nell'ultima settimana
- Solo per le donne: mi trovo in stato di gravidanza*

- CONDIZIONI PSICOFISICHE ATTUALI

- Ritengo attualmente di trovarmi in cattive condizioni psicofisiche
- Se si, perché? ____
- Nella notte precedente ho dormito male/per meno di 6 ore
- Sono appena rientrato da un viaggio transmeridiano (soffro attualmente per il fuso orario della zona in cui mi trovo)
- Nelle ultime 48 ore ci sono stati cambiamenti significativi nel consumo di nicotina o di caffeina
- Se si, quali?

Participants will be included only if they will answer NO in all the questions marked with the asterisk. For the other questions, the experimenters will evaluate the answers. Participants will be asked to communicate to the experimenter any possible change in drug therapy throughout the study. The session will be postponed whenever participants will not be in the optimal conditions. Healthy participants will be recruited at the post-graduate courses hosted by MEDEA or by UNIBG and tested in a structure with medical supervision.

In order to be eligible to participate in WP3 and WP4, CP patients must meet all of the following criteria:

- clinical signs of unilateral or bilateral upper limb deficits;
- MRI confirmed diagnosis according to Surveillance of Cerebral Palsy (SCPE) criteria (26),
- Intelligence quotient (IQ) > 50;
- -MACS level ≤ 4;
- -Spastic hypertonus with Modified Ashwort Scale ≤ 3;
- absence of severe behavioral deficits;
- absence of cochlear implant, cardiac pacemaker, neuro-stimulators, clips, fragments or metal splinters in the brain or skull except for titanium.

A potential patient who meets any of the following criteria will be excluded from participation in this study:

- severe concomitant pathologies independent of CP;
- motor deficits caused by insults other than perinatal ones (e.g. head trauma);
- severe visual impairment that may interfere with treatment or testing;
- treatments for spasticity or functional surgery of the upper limb in the previous 6 months or planned during the duration of the study;
- participation in traditional motor treatment in the 6 months prior to the start of the study;
- Epileptic seizure in the last 2 years.

Numero di pazienti che si prevede arruolare

Total: 220
Wp1 (Medea): 88
Wp2 (UNIBG): 44
Wp3 (Mondino, Medea; ASTT Spedali Civili): 44
Wp4 (ASTT Spedali Civili, Medea, Mondino): 44

Punto di arruolamento

Participants will be considered enrolled after the informed consent will be signed.

Numero di dispositivi medici sperimentali che si prevede di impiegare

2 (tVNS-E and Starstim tDCS)

Durata dello studio

36 months.

Procedure medico-chirurgiche e follow-up di studio

Wp1. For each participant active or control (sham) tVNS will be applied for about 60 minutes during the execution of the computerized visuomotor task. tVNS will be performed by using the CE marked tVNS®*E* device (tVNS technologies GmbH, Erlangen, Germany). It consists in a programmable stimulation unit connected to two titan ear electrodes that are mounted on a gel frame, allowing to generate and transfer electric impulses from the stimulation unit to the surface of the skin, where the electrodes are applied. For active tVNS the stimulation electrode will be applied on the cymba conchae containing the afferent auricular branch of the VN, while for the sham-tVNS electrodes will be applied on the centre of the left lobe auricle area, that is free of cutaneous vagal innervation and whose stimulation does not affect VN activity. The electrodes can be used as an earphone for both real and sham tVNS and the stimulator unit is pocket-sized and can be used during movements. The intensity of tVNS will be set at an intensity corresponding to the perceptual threshold, below the pain threshold and pulses will be delivered every 200–300 µs, at a frequency of 25 Hz. The stimulation will be always applied to the left ear. Following tVNS, different measures of cortical excitability and inhibition will be taken by using Sp and PP TMS protocols. TMS will be delivered over the scalp portion overlying the participant's left or right motor hand region (according to the practicing hand) through a 70-mm-figure-of-eight coils connected to a Magstim 200^2 BiStim (Magstim Company, Carmarthenshire, Wales, UK). Motor Evoked Potentials (MEPs) will be recorded from the contralateral index finger muscle (FDI) with a Biopac MP-36 (BIOPAC Systems, Inc., Goleta, CA). With respect to the the PAS protocol, ulnar nerve stimulation will be delivered with a surface electrode positioned 3 cm above the palmar wrist crease of the arm. The intensity will be set at an intensity corresponding to the 300% of the perceptual threshold (i.e., at the minimum perceivable stimulator intensity, mA). The PAS protocol will comprise 200 pairs of stimuli (TMS pulses and ulnar nerve stimulation) given at 0.25 Hz over 13 minutes. In each stimulation, stimulation of the ulnar nerve will

be followed 25 ms later by a TMS pulse at 130% of the rMT. Blocks of 20 Pre-Post PAS baseline MEPs will be collected for each session.

Wp2. After the recording of 5 min eyes-open resting state cortical activity, participants will perform the computerized visuomotor task during the EEG acquisition. The EEG recordings will be analyzed to detect the individual prominent spectral peak within the gamma bandwidth – or individual gamma frequency (IGF) – emerged in the prefrontal electrodes. The IGF will be selected as the target frequency to be-delivered by means of tACS. Participants will then perform again the computerized visuomotor task (part 2) during the administration of the cerebellar tACS. Immediately after the end of the stimulation, indices of motor excitability and cortical inhibition will be assessed following the same procedure applied in WP1. According to the fronto-cerebellar montage proposed here, the active electrode will be positioned over the right cerebellar hemisphere and the reference electrode will be placed over the left dorsolateral-prefrontal cortex (F3). TACS stimulation will be set at 2 mA and will consist of sinusoidal currents tuned to the IGF (range: 40-60 Hz), with a ramp-up/down phase of 30-s, applied for the entire duration of the visuomotor task, i.e., approximately 15 minutes. A sham control condition, in which the currents are ramp-up/down for 10-s, will be delivered in a separate session. The combined tACS-EEG Starstim 8 System (Neuroelectrics, Barcelona, Spain) will be used.

Wp3. Patients allocated to the tVNS combined motor training arm, will be then allocated to real- or sham-tVNS group. Before starting the rehabilitation program patients will perform with the less affected hand the computerized visuomotor task (tested in WP1-WP2) during the delivery of either active or sham tVNS. The tVNS, active or sham, will be applied starting 15 minutes before the training and for the first hour of its course, throughout the entire rehabilitation treatment of bimanual activities. Active and sham tVNS will be applied as described in WP1. Safety and tolerability outcome of the neuro-stimulation technique will be assessed by measuring vital parameters (i.e., capillary saturation of O2 (SPO2) and HR with the finger pulse oximeter) and by self-questionnaire perception at the beginning and end of stimulation. The efficacy outcome of the stimulation-combined treatment will be evaluated with the clinical scales described in “*Variabili da misurare*” session.

Wp4. Patients allocated to real- or sham-tACS will be then allocated to the real or sham tACS group. Before starting the rehabilitation treatment patients will participate in a one-shot experiment, aimed at identifying the IGF and at examining the online effects of tACS on the computerized visuomotor task tested in WP2. Patients will perform, with the less affected hand, the task during the EEG recording, according to the technical procedure applied in WP2. Subsequently, the EEG recordings will be analyzed to detect the IGF emerged in the prefrontal electrodes. The IGF will be selected as the target frequency to be-delivered by means of tACS. The IGF detected in the one-shot experiment will be selected as the target frequency to be-delivered by means of the tACS during the rehabilitation activities. Active and sham tACS will be applied following the same procedures of Wp2, but with a lower intensity of stimulation (1mA) during performance of the visuomotor task. Tolerability outcomes of the neuro-stimulation technique will be assessed by self-questionnaire perception at the beginning and end of stimulation.

Fattori noti o prevedibili che possono compromettere gli esiti e l'interpretazione dei risultati

Given that efferent vagal fibres connected to the right ear might modulate cardiac function, the tVNS stimulation in Wp1 and Wp3 will be always applied to the left ear, whose stimulation does not exert arrhythmic effects (Farmer et al., 2021).

Phosphene elicitation after tACS in Wp2 and Wp4: cerebellar-tACS-induced phosphenes may interfere with task execution. However, this phenomenon has been observed especially after alpha and beta cerebellar tACS, thus stimulating at the gamma bandwidth should reduce the risk of phosphenes' occurrence; iv) Response variability according to gender of participants: the factor gender will be considered in the analysis in order to examine its potential influence on the effect of tACS.

Interruzione e ritiro dei soggetti dalla indagine clinica

Any individuals who withdraws from the study prematurely will be replaced by a newly recruited participant until the desired sample size will be reached. Participants who will withdraw from the experiment for any reason different than adverse events will not be followed-up. However, in case of an adverse event participants will be followed up until the events have abated, or until a stable situation has been reached. In this last case, follow up may include additional tests and/or referral to the general physician or a medical specialist. In any case, there will be no costs to be borne deriving from participating or interrupting the studies.

Piano di monitoraggio dell'indagine clinica

The 4 partners of BOOST will be coordinated by MEDEA, who will be in charge of supervising at both scientific and administrative levels all the phases of the project. The coordinator will guaranty the harmonization of the protocols for NIBS, functional treatment and evaluation of participants and will support the procedures for data sharing and protection, particularly for the multicentric clinical trial studies. MEDEA and UNIBG will jointly contribute to the design of the tVNS and tACS protocols, in continuous exchange with the clinical partners, and will lead data collection under medical supervision, analysis and dissemination for WP1 and WP2, respectively. Patients for the two arms of the clinical trials will be recruited at MEDEA, MONDINO and SPEDALI CIVILI. The clinical trial of the tVNS-combined motor rehabilitation (WP3) will be led by MEDEA, who will be in charge of centralized data analysis and result dissemination, in collaboration with MONDINO and SPEDALI CIVILI. The clinical trial of the tACS-combined motor rehabilitation (WP4) will be led by MONDINO, who will be, in strict collaboration with UNIBG, in charge of centralized data analysis and result dissemination.

WP1 is estimated to be carried out in the first 12 months of the project, in parallel to WP2. After the approval of the Ethical committee, recruitment and testing of healthy adults for the tVNS experiments will last 10 months and will be completed by month 10 (MS1), leaving the last 2 months for data analysis and dissemination of results. The relative publications are expected to be ready at month 12 (MS2).

WP2 is estimated to be carried out in 12 months, in parallel to WP1. Recruitment and testing of healthy adults for the tACS experiments will last 10 months and will be completed by month 10 (MS1), leaving the last 2 months for data analysis and dissemination of results. The relative publications are expected to be ready at month 12 (MS2).

WP3 is estimated to be carried out in 24 months, in parallel to WP4. Recruitment and testing of patients will last 22 months, leaving the last 2 months for data analysis and dissemination of results. At the end of the second year of the project (month 24, MS3), we expect to have recruited and tested (at least at the first post- treatment session) about 50% of the sample (22 patients). This will be a critical milestone to evaluate the quality of the recruitment procedure and to undertake any corrections. Patient recruitment is expected to be completed by month 34 (MS4) and the relative publication to be ready by month 36 (MS5).

WP4 is estimated to be carried out in 24 months, in parallel to WP3. Recruitment and testing of patients will last 22 months, leaving the last 2 months for data analysis and dissemination of results. At the end of the second year of the project (month 24, MS3), we expect to have recruited and tested (at least at the first post- treatment session) about 50% of the sample (22 patients). This will be a critical milestone to evaluate the quality of the recruitment procedure and to undertake any corrections. Patient recruitment is expected to be completed by month 34 (MS4) and the relative publication to be ready by month 36 (MS5).

The coordinator will organize meetings between the partners every 3 months, for updates on the work progress and for planning any prompt adjustments or redress in case of divergences from the timeline. The coordinator will be also in charge of administrative control, and an external audit will be appointed by the coordinator for all partners. At the end of each year, the coordinator will require the other partners to report on the scientific activities and on the expenses, providing the relative documentation. The GANTT chart of the project is reported below.

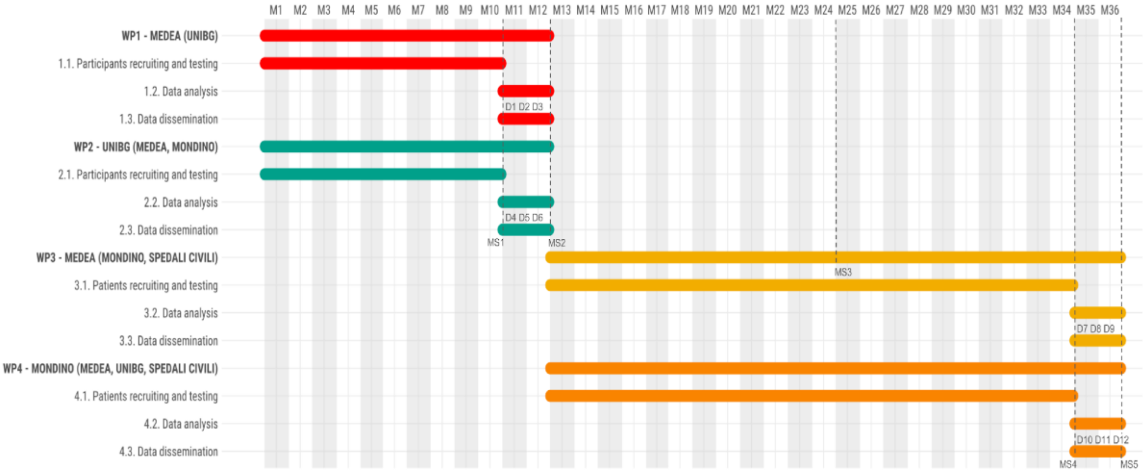

Assicurazione della qualità, procedure di controllo, gestione dei dati e conservazione della documentazione

Personal data will be pseudonymised, by using alphanumeric character so that they could no longer be attributed to a specific participant without the use of additional information. Such information will be stored in specific cabinets in order to protect confidentiality before, during, and after the study (see the Informativa e consenso dati module). Data will be protected and stored for a period of 25 years. Electronic data will be saved on protected electronic data repository. This will be done securely and in compliance with the GDPR policy.

Deviazioni al piano di valutazione clinica

Throughout the project, the PIs will be advised to identify potential risks and suggestions for solutions will be discussed on a regular basis allowing to monitor the scientific progress of the project; to fine tune the analyses and interpretation of findings prior to writing for dissemination as scientific papers. The support offices at the host institutions count on experienced personnel to assist the researchers with the successful implementation and management of the project. In addition to the preventive actions of close project monitoring by the Research Office, a full risk analysis has been conducted and contingency plans have been developed to highlight and preserve each other WP from possible fallbacks and delays and in case of any deviations from the original plan. Amount of persons will be in line with the proposed action, specific objectives and the interdisciplinarity of the action, that need the recruitment of combined expertise of neurologists, psychologists and neuroscientists.

Eventi avversi

Unintended effects of neurostimulation will be carefully checked by asking participants' to fill ad hoc questionnaires (Fertonani et al., 2015; Giustiniani et al., 2022). The questionnaires will be adapted to be understood also by children and adolescents. Hereafter, the risks associated to the NIBS are described.

tVNS. Systematic review analysed the side effects reported on a total of 51 studies published between the 2007 to the 2017 and involving a total of 1322 participants (Redgrave et al., 2018). These studies were conducted on healthy or clinical populations. In particular they focused on the treatment of epilepsy, migraine, tinnitus and depression, or they were conducted on patients with schizophrenia, impaired glucose tolerance, pain, refractory grastroparesis, atrial fibrillation and asthma. The most commonly reported side effects were tactile sensations and skin irritation under the electrode (240 participants, 18.2%), headache (47 participants, 3.6%), vertigo (20 participants, 1.5%), nausea (16 participants; 1.2%), nasopharyngitis (23 participants, 1.7%). The 2.6% of participants dropped out of the studies. A total of 5 studies (7 participants in total) reported cardiac side effects, including palpitations, arrhythmia, hypotension and bradycardia, all effects were transient and asymptomatic). Gastrointestinal side effects (nausea, vomiting) were reported in less than 1.2% of the patients. Overall, 30 serious adverse effects were reported but only 3 of them were considered possibly or probably tVNS related and consisted in palpitations, vestibular neuritis and skin lesion.

tACS. The use of low intensity electric current stimulation has to date never resulted in significant adverse effects, apart from rare cases of mild headache, nausea, dizziness or itching underneath the electrodes (Nitsche et al., 2008). With respect in particular to tACS Splittgerber et al., (2020) investigated the effects of tolerability for tACS in 15 healthy children and adolescents and in a sample of 28 healthy adults. The presence and the severity of headaches, change or difficulties in concentration, mood, visual perception, presence of fatigue, and discomforting sensations like pain, tingling, itching or burning were reported and rated through a numerical analogue scale from one (very mild) to five (extremely high). By considering the effects reported by children participants during tACS, the most commonly reported effect consisted in flickering sensations (40%), general unpleasantness (20%); Tingling (20%), itching and fatigue (13%), pain, nervousness and difficulties in concentration (6%). Headhache and burning sensations were not reported. Overall, the intensity of these sensation was of mild to moderate intensity (from 1 to 2.5 score). In adults, flickering sensations (57%), tingling (28%) fatigue (21%) general unpleasantness (17%); itching (14%) pain and nervousness (10%), burning (7%) and difficulties in concentration (3%) were reported. Headhache was not reported. Overall, the intensity of these sensations was of mild to moderate intensity (from 1 to 2.1 score). In the study by Alon and colleagues (1998) with children (N=7, 2.5-7.5 yo aged) with cerebral palsy, all children tolerated the active stimulation well. There were no reports of seizures, episodes of nausea or vomiting, or sleep disruption by any of the children or their parents. No side effects were mentioned in the study by Prehn-Kristensen et al., (2014), applying slow frequency tACS in 12 children (10-14 yo aged) with Attention Deficit and Hyperactivity Disorder.

TMS. In a recent review (Rossi et al., 2021) an update of the ten-years old safety guidelines for the application of TMS in research and clinical setting (Rossi et al., 2009) has been reported. The update refers also on the possible induction of seizures that is theoretically the most serious risk of TMS. Of note, most of the cases of accidental seizures induced by TMS occurred prior to the definition of safety limits. By considering the large number of subjects and patients who received TMS since 1998 and the small number of seizures, it has become apparent that such a risk is low, even in patients taking drugs acting on the central nervous system, at least with the use of traditional stimulation parameters and focal coils for which large data sets are available. Hearing problems can be induced by the rapid mechanical deformation of the TMS discharging coil. The use of hearing protection (like earplugs) will be ensured. Short-lived low to moderate cognitive effects of TMS have been reported in both healthy and disease, either in the direction of impaired or enhanced functions. With respect to the single pulse SP-TMS or the paired pulse PP-TMS protocols, side effects have been revised in 2009 (Rossi et al., 2009). Seizure induction is considered as rare for single pulse TMS; while it was never reported for PP-TMS. Syncope has been considered a possible epiphenomenon either during SP or PP-TMS. Possible, and likely possible but not reported are transient headache, local pain, neck pain, toothache, paraesthesia and transient hearing changes during SP or PP-TMS. No other effects were reported or considered as possible by the consensus group. With respect to PAS protocol, no major Adverse Effects including seizure occurrence was reported in the studies revised for the 2021 update (Rossi et al., 2021) in both healthy subjects and pathological conditions. Thus, there should not be any special concern in studies of this type.

Of note, the use of the devices for neurostimulation will be carried out under medical supervision. All studies will be performed in clinically supervised facilities. The screening, the recruitment and the treatment of the patients will be supervised by the referring physician.

All serious adverse effect will be reported via e-mail to the Etic committee that approved the protocol, within 15 days after the promotor has first knowledge of the serious adverse reactions. SAEs that result in death or are life threatening should be reported expeditedly. The expedited reporting will occur not later than 7 days after the responsible investigator has first knowledge of the adverse reaction. This is for a preliminary report with another 7 days for completion of the report.

Emendamenti al piano di valutazione clinica

If amendments to the protocol will be necessary during the study, the changes will be submitted for approval of the Ethics Committee. The changes will be not applied until having received favourable opinion from the Ethics Committee.

Termine anticipato e sospensione della valutazione clinica

The study will be terminated when serious adverse event occurs. All recruited patients, however, will receive follow-up evaluation as intended in the initial protocol.

Statistica

Wp1: For each variable, a 2x3x2 mixed model ANOVA will be conducted with the within-subjects variables hand (dominant vs. nondominat) and tVNS condition (baseline vs. real- vs. sham-tVNS) and gender as a between-subject variable. In the case of a violation of the sphericity assumption (Mauchly's tests), a Greenhouse-Geisser correction will be used before interpreting the results. In case of significant main effects or interactions, post-hoc analysis will be conducted with multiple comparison test correction methods. Significance threshold will be set as $p<0.05$ (two-tailed). For each of the two studies of Wp1, sample size for this design (numerator df = 2) has been determined using GPower 3 to detect, at a significance threshold of 0.05, moderate to large effects (f (U) = 0.35) with a power of 0.80 (total sample size = 44).

Wp2: Separate 2x3x2 mixed model ANOVA will be conducted to evaluate changes in behavioral performance, as well as physiological indices with the within-subjects variables hand (dominant vs. non dominant) and tACS condition (baseline vs. IGF vs. sham tACS) and gender as between-subject factor. In the case of a violation of the sphericity assumption (Mauchly's tests), a Greenhouse-Geisser correction will be used before interpreting the results. In case of significant main effects or interactions, post-hoc analysis will be conducted with multiple comparison test correction methods. Significance threshold will be set as $p<0.05$ (two-tailed). Sample size for this design (numerator df = 2) has been determined using GPower 3 to detect, at a significance threshold of 0.05, moderate to large effects (f (U) = 0.35) with a power of 0.80 (22 per group).

Wp3 and Wp4: Outcome measures will be analyzed with mixed model 2x3 ANOVAs, with group (active vs. sham) as between-subject factor and time (T0, T1, T2) as within-subject variable. Using the GPower 3 software, we estimated that this design (numerator DF=2) requires, assuming a significance threshold of 0.05, a sample of 22 subjects per group to detect moderate to large effects (η2p = 0.1, f (U) = 0.35), with a power of 0.80.

A modified intention-to-treat analysis approach will be adopted, including in the analyses all the participants who had completed the pre- and post-treatment evaluation sessions, even if they have not completed all the training sessions. No imputation of missing data, however, will be used considering the limited sample size and observation points.

Politica di pubblicazione dei dati

Results will be published in peer review indexed journals, with an open-access policy/option. Preliminary versions of the research papers will be made publicly available through the working paper series published on the project website. The partners will adhere to RRI principles of *open access*, with 100% publications in open access journals or with open-access option. We will adhere to principles of transparency and reproducibility in research by preregistering the clinical trials and sharing research material and results of all studies in public repositories (https://osf.io/). The partners will promote data integrity, management and protection (established procedures are available in all partners' institutions and will be shared).

Bibliografia

Alon, G., Syron, S. C., & Smith, G. V. (1998). Is Transcranial Electrical Stimulation (TCES) a Safe Intervention for Children with Cerebral Palsy? *Neurorehabilitation and Neural Repair*, *12*(2), 65–71. https://doi.org/10.1177/154596839801200204

Antal, A., Alekseichuk, I., Bikson, M., Brockmöller, J., Brunoni, A. R., Chen, R., Cohen, L. G., Dowthwaite, G., Ellrich, J., Flöel, A., Fregni, F., George, M. S., Hamilton, R., Haueisen, J., Herrmann, C. S., Hummel, F. C., Lefaucheur, J. P., Liebetanz, D., Loo, C. K., … Paulus, W. (2017). Low intensity transcranial electric stimulation: Safety, ethical, legal regulatory and application guidelines. *Clinical Neurophysiology: Official Journal of the International Federation of Clinical Neurophysiology*, *128*(9), 1774–1809. https://doi.org/10.1016/j.clinph.2017.06.001

Assenza, G., Campana, C., Colicchio, G., Tombini, M., Assenza, F., Di Pino, G., & Di Lazzaro, V. (2017). Transcutaneous and invasive vagal nerve stimulations engage the same neural pathways: In-vivo human evidence. *Brain Stimulation*, *10*(4), 853–854. https://doi.org/10.1016/j.brs.2017.03.005

Capone, F., Assenza, G., Di Pino, G., Musumeci, G., Ranieri, F., Florio, L., Barbato, C., & Di Lazzaro, V. (2015). The effect of transcutaneous vagus nerve stimulation on cortical excitability. *Journal of Neural Transmission*, *122*(5), 679–685. https://doi.org/10.1007/s00702-014-1299-7

Cioni, G., D'Acunto, G., & Guzzetta, A. (2011). Perinatal brain damage in children: Neuroplasticity, early intervention, and molecular mechanisms of recovery. *Progress in Brain Research*, *189*, 139–154. https://doi.org/10.1016/B978-0-444-53884-0.00022-1

Dawson, J., Liu, C. Y., Francisco, G. E., Cramer, S. C., Wolf, S. L., Dixit, A., Alexander, J., Ali, R., Brown, B. L., Feng, W., DeMark, L., Hochberg, L. R., Kautz, S. A., Majid, A., O'Dell, M. W., Pierce, D., Prudente, C. N., Redgrave, J., Turner, D. L., … Kimberley, T. J. (2021). Vagus nerve stimulation paired with rehabilitation for upper limb motor function after ischaemic stroke (VNS-REHAB): A randomised, blinded, pivotal, device trial. *The Lancet*, *397*(10284), 1545–1553. https://doi.org/10.1016/S0140-6736(21)00475-X

Duarte, N. de A. C., Grecco, L. A. C., Galli, M., Fregni, F., & Oliveira, C. S. (2014). Effect of transcranial direct-current stimulation combined with treadmill training on balance and functional performance in children with cerebral palsy: A double-blind randomized controlled trial. *PloS One*, *9*(8), e105777. https://doi.org/10.1371/journal.pone.0105777

Farmer, A. D., Strzelczyk, A., Finisguerra, A., Gourine, A. V., Gharabaghi, A., Hasan, A., Burger, A. M., Jaramillo, A. M., Mertens, A., Majid, A., Verkuil, B., Badran, B. W., Ventura-Bort, C., Gaul, C., Beste, C., Warren, C. M., Quintana, D. S., Hämmerer, D., Freri, E., … Koenig, J. (2021). International Consensus Based Review and Recommendations for Minimum Reporting Standards in Research on Transcutaneous Vagus Nerve Stimulation (Version 2020). *Frontiers in Human Neuroscience*, *14*, 568051. https://doi.org/10.3389/fnhum.2020.568051

Fertonani, A., Ferrari, C., & Miniussi, C. (2015). What do you feel if I apply transcranial electric stimulation? Safety, sensations and secondary induced effects. *Clinical Neurophysiology: Official Journal of the International Federation of Clinical Neurophysiology*, *126*(11), 2181–2188. https://doi.org/10.1016/j.clinph.2015.03.015

Finisguerra, A., Borgatti, R., & Urgesi, C. (2019). Non-invasive Brain Stimulation for the Rehabilitation of Children and Adolescents With Neurodevelopmental Disorders: A Systematic Review. *Frontiers in Psychology*, *10*, 135. https://doi.org/10.3389/fpsyg.2019.00135

Fleming, M. K., Theologis, T., Buckingham, R., & Johansen-Berg, H. (2018). Transcranial direct current stimulation for promoting motor function in cerebral palsy: A review. *Journal of NeuroEngineering and Rehabilitation*, *15*(1), 121. https://doi.org/10.1186/s12984-018-0476-6

Giustiniani, A., Vallesi, A., Oliveri, M., Tarantino, V., Ambrosini, E., Bortoletto, M., Masina, F., Busan, P., Siebner, H. R., Fadiga, L., Koch, G., Leocani, L., Lefaucheur, J. P., Rotenberg, A., Zangen, A., Violante, I. R., Moliadze, V., Gamboa, O. L., Ugawa, Y., … Burgio, F. (2022). A questionnaire to collect unintended effects of transcranial magnetic stimulation: A consensus based approach. *Clinical Neurophysiology: Official Journal of the International Federation of Clinical Neurophysiology*, *141*, 101–108. https://doi.org/10.1016/j.clinph.2022.06.008

Grecco, L. A. C., de Almeida Carvalho Duarte, N., Mendonça, M. E., Cimolin, V., Galli, M., Fregni, F., & Santos Oliveira, C. (2014). Transcranial direct current stimulation during treadmill training in children with cerebral palsy: A randomized controlled double-blind clinical trial. *Research in Developmental Disabilities*, *35*(11), 2840–2848. https://doi.org/10.1016/j.ridd.2014.07.030

Jackman, M., Lannin, N., Galea, C., Sakzewski, L., Miller, L., & Novak, I. (2020). What is the threshold dose of upper limb training for children with cerebral palsy to improve function? A systematic review. *Australian Occupational Therapy Journal*, *67*(3), 269–280. https://doi.org/10.1111/1440-1630.12666

Koenig, J., Rash, J. A., Campbell, T. S., Thayer, J. F., & Kaess, M. (2017). A Meta-Analysis on Sex Differences in Resting-State Vagal Activity in Children and Adolescents. *Frontiers in Physiology*, *8*, 582. https://doi.org/10.3389/fphys.2017.00582

Krishnan, C., Santos, L., Peterson, M. D., & Ehinger, M. (2015). Safety of noninvasive brain stimulation in children and adolescents. *Brain Stimulation*, *8*(1), 76–87. https://doi.org/10.1016/j.brs.2014.10.012

Kuo, H.-C., Litzenberger, J., Nettel-Aguirre, A., Zewdie, E., & Kirton, A. (2022). Exploring Clinical and Neurophysiological Factors Associated with Response to Constraint Therapy and Brain Stimulation in Children with Hemiparetic Cerebral Palsy. *Developmental Neurorehabilitation*, *25*(4), 229–238. https://doi.org/10.1080/17518423.2021.1964103

Lee, C. M., & Bo, J. (2021). Visuomotor adaptation and its relationship with motor ability in children with and without autism spectrum disorder. *Human Movement Science*, *78*, 102826. https://doi.org/10.1016/j.humov.2021.102826

Manto, M., Bower, J. M., Conforto, A. B., Delgado-García, J. M., da Guarda, S. N. F., Gerwig, M., Habas, C., Hagura, N., Ivry, R. B., Mariën, P., Molinari, M., Naito, E., Nowak, D. A., Oulad Ben Taib, N., Pelisson, D., Tesche, C. D., Tilikete, C., & Timmann, D. (2012). Consensus paper: Roles of the cerebellum in motor control--the diversity of ideas on cerebellar involvement in movement. *Cerebellum (London, England)*, *11*(2), 457–487. https://doi.org/10.1007/s12311-011-0331-9

Marrosu, F., Serra, A., Maleci, A., Puligheddu, M., Biggio, G., & Piga, M. (2003). Correlation between GABA(A) receptor density and vagus nerve stimulation in individuals with drug-resistant partial epilepsy. *Epilepsy Research*, *55*(1–2), 59–70.

Meyers, E. C., Solorzano, B. R., James, J., Ganzer, P. D., Lai, E. S., Rennaker, R. L., Kilgard, M. P., & Hays, S. A. (2018). Vagus Nerve Stimulation Enhances Stable Plasticity and Generalization of Stroke Recovery. *Stroke*, *49*(3), 710–717. https://doi.org/10.1161/STROKEAHA.117.019202

Naro, A., Bramanti, A., Leo, A., Manuli, A., Sciarrone, F., Russo, M., Bramanti, P., & Calabrò, R. S. (2017). Effects of cerebellar transcranial alternating current stimulation on motor cortex excitability and motor function. *Brain Structure & Function*, *222*(6), 2891–2906. https://doi.org/10.1007/s00429-016-1355-1

Naro, A., Leo, A., Russo, M., Cannavò, A., Milardi, D., Bramanti, P., & Calabrò, R. S. (2016). Does Transcranial Alternating Current Stimulation Induce Cerebellum Plasticity? Feasibility, Safety and Efficacy of a Novel Electrophysiological Approach. *Brain Stimulation*, *9*(3), 388–395. https://doi.org/10.1016/j.brs.2016.02.005

Nitsche, M. A., Cohen, L. G., Wassermann, E. M., Priori, A., Lang, N., Antal, A., Paulus, W., Hummel, F., Boggio, P. S., Fregni, F., & Pascual-Leone, A. (2008). Transcranial direct current stimulation: State of the art 2008. *Brain Stimulation*, *1*(3), 206–223. https://doi.org/10.1016/j.brs.2008.06.004

O'Leary, G. H., Jenkins, D. D., Coker-Bolt, P., George, M. S., Kautz, S., Bikson, M., Gillick, B. T., & Badran, B. W. (2021). From adults to pediatrics: A review noninvasive brain stimulation (NIBS) to facilitate recovery from brain injury. In *Progress in Brain Research* (Vol. 264, pagg. 287–322). Elsevier. https://doi.org/10.1016/bs.pbr.2021.01.019

Park, H.-J., Kim, C. H., Park, E. S., Park, B., Oh, S. R., Oh, M.-K., Park, C. I., & Lee, J. D. (2013). Increased GABA-A Receptor Binding and Reduced Connectivity at the Motor Cortex in Children with Hemiplegic Cerebral Palsy: A Multimodal Investigation Using ^{18} F-Fluoroflumazenil PET,

Immunohistochemistry, and MR Imaging. *Journal of Nuclear Medicine*, *54*(8), 1263–1269. https://doi.org/10.2967/jnumed.112.117358

Prehn-Kristensen, A., Munz, M., Göder, R., Wilhelm, I., Korr, K., Vahl, W., Wiesner, C. D., & Baving, L. (2014). Transcranial Oscillatory Direct Current Stimulation During Sleep Improves Declarative Memory Consolidation in Children With Attention-deficit/hyperactivity Disorder to a Level Comparable to Healthy Controls. *Brain Stimulation*, *7*(6), 793–799. https://doi.org/10.1016/j.brs.2014.07.036

Redgrave, J., Day, D., Leung, H., Laud, P. J., Ali, A., Lindert, R., & Majid, A. (2018). Safety and tolerability of Transcutaneous Vagus Nerve stimulation in humans; a systematic review. *Brain Stimulation*, *11*(6), 1225–1238. https://doi.org/10.1016/j.brs.2018.08.010

Rossi, S., Antal, A., Bestmann, S., Bikson, M., Brewer, C., Brockmöller, J., Carpenter, L. L., Cincotta, M., Chen, R., Daskalakis, J. D., Di Lazzaro, V., Fox, M. D., George, M. S., Gilbert, D., Kimiskidis, V. K., Koch, G., Ilmoniemi, R. J., Lefaucheur, J. P., Leocani, L., … basis of this article began with a Consensus Statement from the IFCN Workshop on «Present, Future of TMS: Safety, Ethical Guidelines», Siena, October 17-20, 2018, updating through April 2020. (2021). Safety and recommendations for TMS use in healthy subjects and patient populations, with updates on training, ethical and regulatory issues: Expert Guidelines. *Clinical Neurophysiology: Official Journal of the International Federation of Clinical Neurophysiology*, *132*(1), 269–306. https://doi.org/10.1016/j.clinph.2020.10.003

Rossi, S., Hallett, M., Rossini, P. M., & Pascual-Leone, A. (2009). Safety, ethical considerations, and application guidelines for the use of transcranial magnetic stimulation in clinical practice and research. *Clinical Neurophysiology*, *120*(12), 2008–2039. https://doi.org/10.1016/j.clinph.2009.08.016

Sadowska, M., Sarecka-Hujar, B., & Kopyta, I. (2020). Cerebral Palsy: Current Opinions on Definition, Epidemiology, Risk Factors, Classification and Treatment Options. *Neuropsychiatric Disease and Treatment*, *16*, 1505–1518. https://doi.org/10.2147/NDT.S235165

Splittgerber, M., Suwelack, J. H., Kadish, N. E., & Moliadze, V. (2020). The Effects of 1 mA tACS and tRNS on Children/Adolescents and Adults: Investigating Age and Sensitivity to Sham Stimulation. *Neural Plasticity*, *2020*, 8896423. https://doi.org/10.1155/2020/8896423

Ulloa, J. L. (2021). The Control of Movements via Motor Gamma Oscillations. *Frontiers in Human Neuroscience*, *15*, 787157. https://doi.org/10.3389/fnhum.2021.787157

Warren, C. M., Tona, K. D., Ouwerkerk, L., van Paridon, J., Poletiek, F., van Steenbergen, H., Bosch, J. A., & Nieuwenhuis, S. (2019). The neuromodulatory and hormonal effects of transcutaneous vagus nerve stimulation as evidenced by salivary alpha amylase, salivary cortisol, pupil diameter, and the P3 event-related potential. *Brain Stimulation*, *12*(3), 635–642. https://doi.org/10.1016/j.brs.2018.12.224

Wessel, M. J., Draaisma, L. R., & Hummel, F. C. (2022). Mini-review: Transcranial Alternating Current Stimulation and the Cerebellum. *Cerebellum (London, England)*. https://doi.org/10.1007/s12311-021-01362-4
